# The Oxepane Motif in Marine Drugs

**DOI:** 10.3390/md15110361

**Published:** 2017-11-15

**Authors:** Héctor Barbero, Carlos Díez-Poza, Asunción Barbero

**Affiliations:** 1 GIR MIOMeT, IU CINQUIMA/Inorganic Chemistry, University of Valladolid, Campus Miguel Delibes, 47011 Valladolid, Spain; hector.barbero@uva.es; 2 Department of Organic Chemistry, University of Valladolid, Campus Miguel Delibes, 47011 Valladolid, Spain; carlos.diez@alumnos.uva.es

**Keywords:** marine drugs, oxepanes, total synthesis, biological activity

## Abstract

Oceans have shown to be a remarkable source of natural products. The biological properties of many of these compounds have helped to produce great advances in medicinal chemistry. Within them, marine natural products containing an oxepanyl ring are present in a great variety of algae, sponges, fungus and corals and show very important biological activities, many of them possessing remarkable cytotoxic properties against a wide range of cancer cell lines. Their rich chemical structures have attracted the attention of many researchers who have reported interesting synthetic approaches to these targets. This review covers the most prominent examples of these types of compounds, focusing the discussion on the isolation, structure determination, medicinal properties and total synthesis of these products.

## 1. Introduction

More than 70% of Earth surface is covered by water, 96.5% of which is found in the oceans. This means that the planet’s largest habitat is the ocean, which is the ecosystem where the major part of animals and plants of Earth lives. This includes microscopic algae, marine plants with roots, sponges, corals and all types of fishes, within others. These marine organisms are a great source of natural products with important biological activities.

The continuous search for compounds with pharmacological properties is one of the main aims of scientists. Natural products have always provided a great contribution to medicine since the first discovery of drugs with positive impact in human health. For thousands of years the common sources of natural products with potential biological activities have been microorganisms and land plants. The search for natural products in the sea is much recent, since a parallel development of the appropriate technology was needed. However, since the 70’s the number of isolated marine natural products with outstanding biological properties has grown enormously [1]. 

A group of marine natural products that attract special interest is polyfunctionalized cyclic ethers. Within them, natural products containing an oxepanyl moiety have been frequently found in sponges, corals, or different marine fungus, within others. Their interesting biological properties include anticancer, antibacterial or antifungal activities. From a structural point of view, many of them are terpenes presenting a great diversity of rings and chains bonded to the oxepanyl ring. 

Their challenging structure, together with their promising medicinal properties, has prompted many researchers to try to develop synthetic methodologies to access this class of marine drugs.

In this review, we try to cover marine drugs that contain a single oxepanyl ring in their structure. However, the coverage will not be comprehensive, since we intend to provide a general overview of the interesting biological, structural and synthetic possibilities of this class of metabolites. Within others, we have omitted the family of lauroxanes, since it has already been reviewed by Fujiwara [2]. We also omitted classical examples of marine polyether biotoxins, such as brevetoxins, ciguatoxins, gambierol, etc., which have been addressed by Nicolau [3]. Some of them, along with abudinol B, brevenal, armatol A, enshuol and others, have been recently reviewed by Jamison [4]. Toxicols, adociasulfates and the latest example of halicloic acids A and B are also interesting substances and appear in a recent review [5]. Of course, we could mention many other families and examples of marine natural products that bear the oxepane moiety (spiroxins, phomactins, clavulazols, etc.) but it is out of the scope of our work. On the other hand, some isolated examples of the compounds described here are also present in other reviews devoted to marine triterpenes [6] or marine natural products [7].

This review is roughly organized in five sections, according to the structure and biogenetic origin of the compounds. For some of the families, only structure determination, isolation and biological activity have been reported. For the rest of the products some of the most relevant synthetic approaches reported till date are described.

## 2. Halogenated Sesquiterpenoids: Aplysistatin and Palisadins

Aplysistatin was first isolated from the South Pacific Ocean sea hare *Aplysia angasi* in 1977 [8]. In his paper, Pettit pointed out that this compound could be derived from its diet, since it is known that sea hares usually graze algae and other similar metabolites had already been isolated from algae [9,10,11,12]. This hypothesis was confirmed in 1980, when aplysistatin was collected from the alga *Laurencia* cf. *palisada* Yamada in 1980 [13]. Since then, it has been identified in many other *Laurencia* species, namely *Laurencia filiformis* [14], *Laurencia implicata* [15], *Laurencia flexilis* [16], *Laurencia karlae* [17], *Laurencia luzonensis* [18], *Laurencia saitoi* [19], *Laurencia similis* [20] and *Laurencia snackeyi* [21].

**Figure 1:**
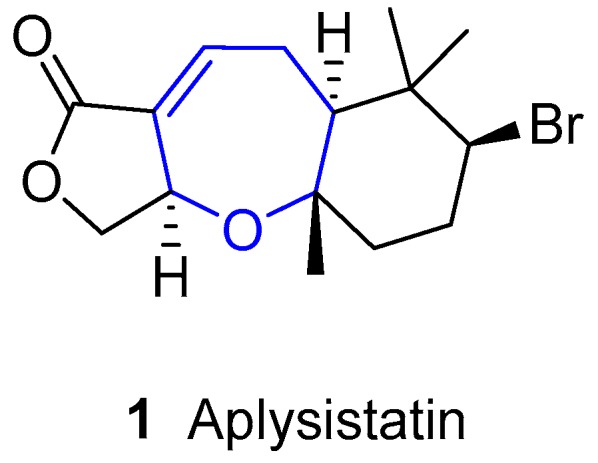
Structure of aplysistatin.

This sesquiterpene bears a unique structure, as shown in Figure 1. Its absolute configuration was established by X-ray diffraction [8,22], showing a *trans*-*anti* stereochemistry for the fused rings. 

As for its biological activity, it was reported to inhibit progression of murine lymphocytic leukemia P-388 (T/C 175 at 400 mg/kg) [8] and a broad range of other cultured tumor cells. It also showed antimalarial activity [23], anti-inflammatory activity and ability to suppress the expressions of iNOS and COX-2 enzymes [24].

**Figure 2:**
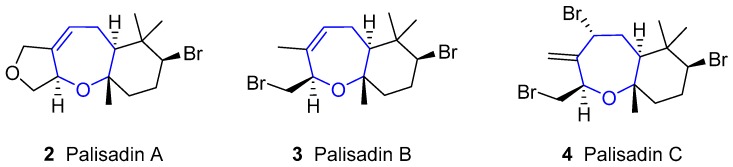
Main structures in the palisadin family.

In 1980, Fenical reported the isolation of palisadin A, palisadin B and other three related compounds from *Laurencia* cf. *palisada* [13]. Later, palisadin C [16], along with other substituted palisadins, has been isolated (structures of this family are shown in Figure 2). They have a similar structure to aplysistatin, although lacking the lactone ring, and are also present in many algae of the *Laurencia* species.

**Figure 3:**
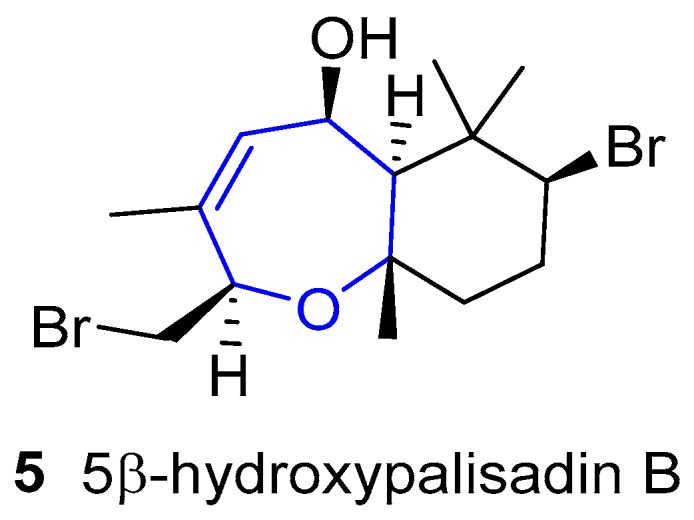
Structure of 5β-hydroxypalisadin B.

5β-hydroxipalisadin B (Figure 3) showed effective anti-inflammatory properties, reducing stress-induced reactive oxygen species formation, and inhibiting the lipopolysaccharide-induced NO production in zebrafish embryos [25].

**Scheme 1:**
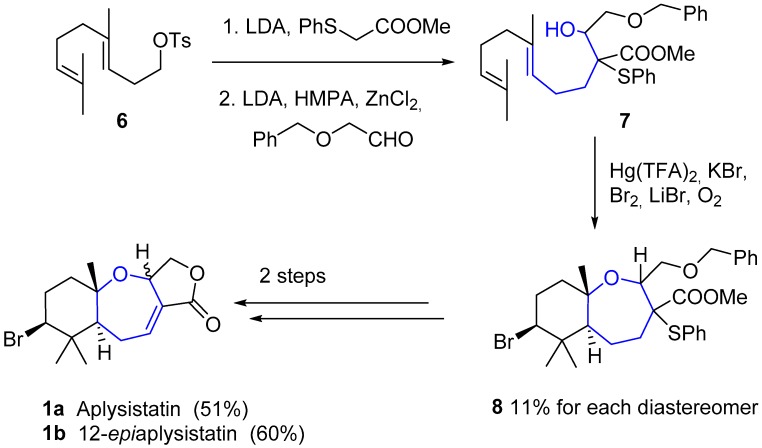
Overview of Hoye’s synthesis of (±)-aplysistatin.

The unusual, but not exceptionally complex, structure of these compounds has attracted the attention of synthetic chemists. In 1979, just two years after its isolation, Hoye and coworkers reported the first total synthesis of aplysistatin [26]. Diene **7** was obtained in two steps from the p-toluenesulfonate ester of homogeraniol **6**, by alkylation with the enolate anion of 2-phenyltioacetate and subsequent aldol condensation. An additional key Hg(TFA)_2_/bromine-mediated cyclization provided a mixture of diastereomeric oxepins **8**, which in two steps (oxidative elimination and final debenzylation-lactonization) provided aplysistatin (**1a**) and 12-*epi*-aplysistatin (**1b**) (see Scheme 1). In the next few years some other total syntheses of **1** were reported which did not imply any improvements in either yields or stereoselectivities [27,28,29,30,31,32]. 

**Scheme 2:**
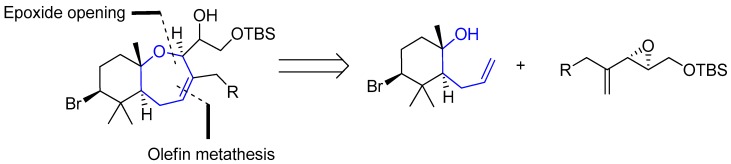
Couladouros’ retrosynthetic analysis of his key intermediate.

Regarding palisadins, Yamashita’s group performed the first total synthesis of (+)-palisadin A and (+)-hydroxypalisadin B [33], also relying on a Hg(TFA)_2_/bromine-mediated cyclization as the key step. In 2004 Couladouros’ group published a general approach to *trans*-fused oxepene-cyclogeranyl systems, and applied it to the synthesis of aplysistatin, palisadins A and B and 12-hydroxypalisadin B [34]. Their strategy followed an alternative route based on the formation of the oxepanyl derivative by addition of a tertiary alcohol to a 1,2-disubstituted epoxide and subsequent ring closing metathesis Scheme 2. 

**Scheme 3:**
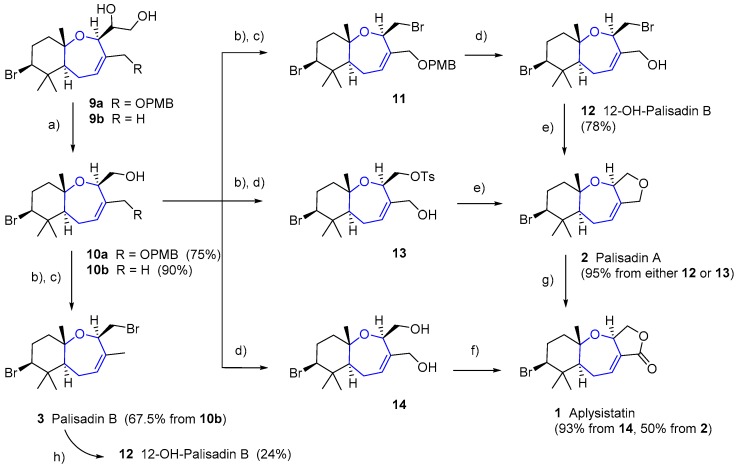
Couladouros’ synthesis of (−)-Aplysistatin, (+)-Palisadin A, (+)-Palisadin B, and (+)-12-hydroxy-Palisadin B. (**a**) NaIO_4_; then NaBH_4_; (**b**) TsCl; (**c**) LiBr, HMPA; (**d**) DDQ; (**e**) K_2_CO_3_; (**f**) MnO_2_; (**g**) PCC; (**h**) SeO_2_, NaBH_4_.

This oxepene intermediate provided easy access to the desired natural products in three or four additional steps, as depicted in Scheme 3.

## 3. Marine Diterpenes: Oxepin Lobatrienol

**Figure 4:**
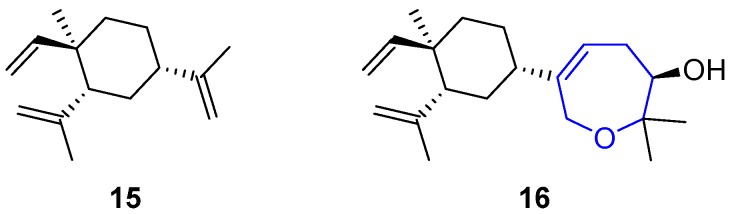
Chemical structures of β-elemene (**19**), common scaffold for all lobanes; and of oxepin lobatrienol (**20**) with oxepine structure highlighted.

Lobanes are a family of diterpenes from genus *Lobophytum*, also known as “devil’s hand corals”. Their habitat covers the shallow waters of Indo-Pacific coasts [35,36], although these compounds have been also isolated from *Eunicea fusca* [37,38], *Sinularia* [39,40,41] and *Sarcophyton* [42] species. Their structure is based on the main scaffold of β-elemene and one of the members of this family, the one called oxepin lobatrienol, possess an oxepane-like moiety (see Figure 4). In such molecule, an oxepanol moiety has substituted a prop-1-en-2-yl unit in C4 [43]. Despite their remarkable bioactivity, few efforts have been devoted to the synthesis of this kind of compounds [44,45,46,47].

All members of lobane family are lethal against *Cladosporium cucumerinum*, a pathogen fungus responsible for scab disease that affects cucumbers, and against genus *Artemia* species (commonly known as brine shrimps). Apparently, the nature of the isoprenyl fragment attached to the basic structure of β-elemene determines which of the two properties predominates. It seems that when the activity against the fungus is increased, the activity against brine shrimps is decreased, and vice versa. These findings suggest a non-generic toxicity and a specific mode of action is yet to be discovered. Interestingly, oxepin lobatrienol possess intermediate activity and it performs well in both tasks, demonstrating, once more, the importance of oxepane-like structures. 

## 4. Marine Triterpenes

This huge group consists of a set of triterpenes bearing oxepane moieties [48,49]. It is believed that their biosynthesis comes from the metabolism of very simple squalene building blocks. They have been named according to their origin, either from the species or the place where they were obtained. Bearing in mind the complexity of the classification, we decided to divide it into 5 categories.

### 4.1. Sipholenols Family

The first compound of this group (sipholenol A) was isolated by Shmueli, Kashman and coworkers [50] from the colonial tube-sponge *Siphonochalina siphonella*, a species whose habitat is the Red Sea. Just two years later, up to nine molecules were already known [51,52]. Less attention was paid in the 90s (mainly due to the discovery of similar families such as sodwanones) until the beginning of the 21st century, when interest for these species was renewed. Nowadays, the number of compounds belonging to sipholenols has increased up to thirty [53,54,55].

**Figure 5:**
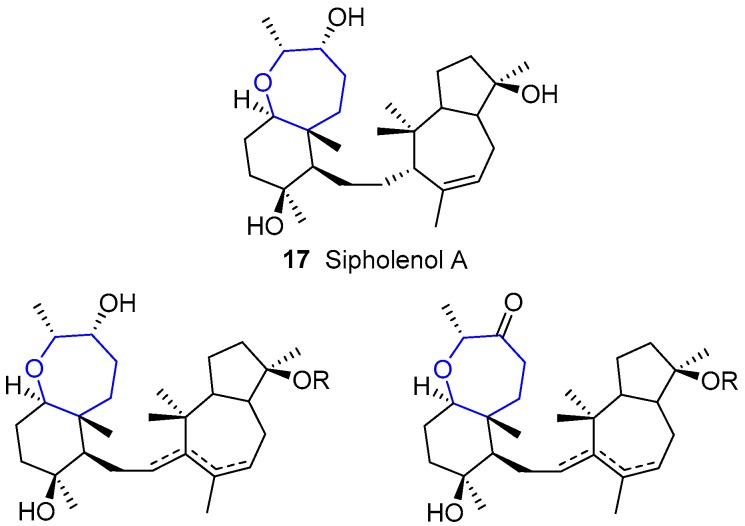
Molecular structures of sipholenol A and the two main scaffolds in the sipholenol family.

From the structural point of view, the molecules contain a hydroazulene fragment (with variable insaturations) linked through an ethylene bridge to a trans-decahydrobenzoxepin, as seen in Figure 5. It must be noted that this is the most common scaffold, but not present in all of them. There are some differences in the last set of discovered molecules, e.g., the rearrangement of azulene fragment or the substitution of an oxepane by a tetrahydropyran.

These molecules have shown good anti-cancer properties [56]. In more detail, they are able to reverse P-glycoprotein-mediated multi drug resistance in cancer cells by inhibiting the drug efflux from this protein, along with other effects [57,58,59,60]. Currently, their antiproliferative effects towards human hepatic and colorectal cancer cells are being studied [61].

Moreover, this family can be used as antifouling agent owing to its ability to disrupt settlement of barnacle larvae.

### 4.2. Neviotanes and Dahabanes

**Figure 6:**
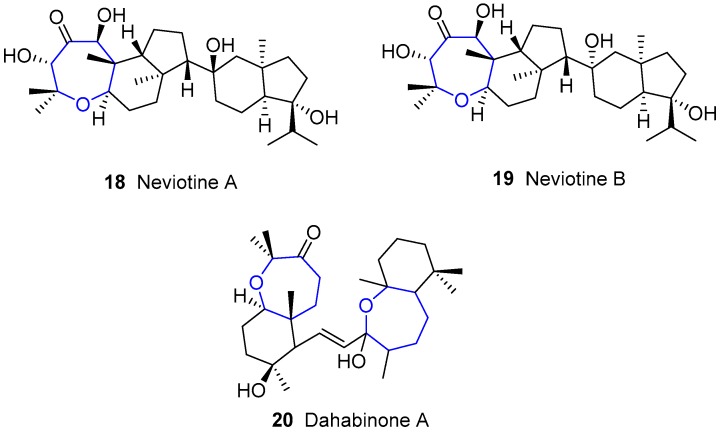
Neviotanes and Dahabinone A discovered by Kashman during their studies with *Siphonochalina siphonella*.

Along with sipholenols, Kashman and collaborators also reported the isolation of Neviotines A and B with Dahabinone A from the same sponge *Siphonochalina siphonella*. Such names have their origin in the place from which these species were extracted: Nevi’ot (Israel) and Dahlak islands (Erithrea), respectively [53,62]. The structure of the latter compound is very similar to that from sipholenols family, bearing a second oxepane unit fused to a cyclohexane instead of the azulene moiety. Unlike sipholenols and dahabanes, neviotanes possess a pentacyclic core in which there are two groups connecting though a single bond (Figure 6). 

In addition, new compounds of this family have been reported recently [63].

### 4.3. Sodwanones

This class of compounds was first isolated from the Indo-Pacific fan sponge *Axinella weltneri*, collected during the summer of 1992 in Sodwana Bay, South Africa [64,65]. Since then, they have been found in other species, namely *Ptilocaulis spiculifer* [66], and *Axinella* cf. *bidderi* [67], and so far they form a family of more than 20 compounds [68,69,70,71,72]. They are examples of the rare marine triterpenes, which have a common structure showing an oxepane-cycloalkane moiety. As far as we know, no attempts of synthesis of any of the sodwanones have been done.

**Figure 7:**
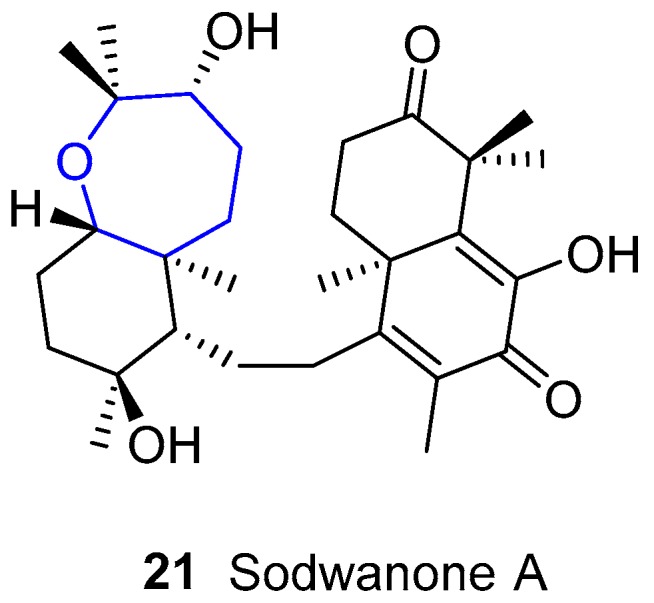
Structure of Sodwanone A.

Many of the sodwanones have shown interesting properties. Sodwanone A (Figure 7) was active against several cell lines, such as a lung carcinoma cell line [67], three esophageal cancer cell lines [73], an ovarian cancer one [71] and some others. Sodwanones G, H and I were found to be toxic against several cancer cell lines [69]. Sodwanone M was reported to be cytotoxic to P-388 murine leukemia cells [68]. Sodwanone S was moderately active against several lines [71] and some other members of the sodwanone family inhibited hypoxia-induced HIF-1 activation in breast and prostate tumor cells [72].

### 4.4. Shaagrockols

**Figure 8:**
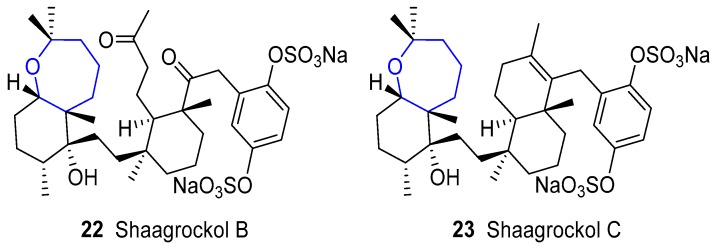
Shaagrockols isolated by Kashman’s group.

In parallel to the discoveries of sodwanones, two new compounds were reported by Kashman’s group in 1992 called Shaagrockols B and C from the Red Sea sponge *Toxiclona toxius* [74,75]. Their name comes from the place where the sponge was collected: Shaag rock, in the entrance to the Gulf of Suez. Structurally, they are very closely related to sodwanones and the main difference arises from the additional tetrahydroquinone fragment that bears sulfonate groups [76,77], as depicted in Figure 8. They have shown antifungal activity against *Candida albicans* and inhibition of Human Immunodeficiency Virus Type 1 Reverse Transcriptase (HIV-1 RT).

In recent years, new members of this family have appeared [78] and some synthetic approaches have been carried out [79].

### 4.5. Raspacionins

**Figure 9:**
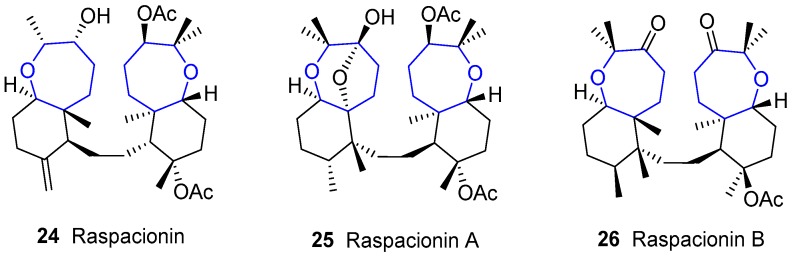
Structures of early raspacionins reported by Puliti and Madaio.

This family was firstly discovered by Puliti, Madaio and collaborators between 1991 and 1992. Compounds were isolated from red encrusting sponge *Raspaciona aculeata* [80,81,82,83]. From the structural point of view, these quasi-symmetrical molecules resemble dahabinone as they contain two oxepane subunits (Figure 9). After such initial discoveries, other minor secondary metabolites were found [84,85].

## 5. Meroterpenoids: Austalides

**Figure 10:**
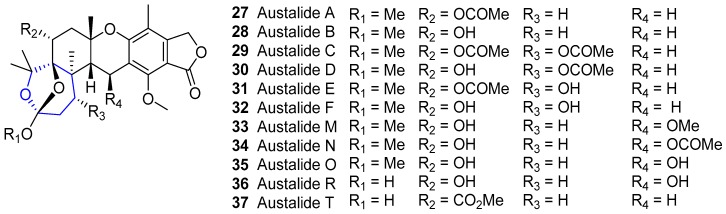
The whole family of oxepane-containing austalides.

This family consists of a 28-membered group of molecules whose origin comes from *Penicillium* and *Aspergillus* marine-derived genera. First discovered in 1981 by Vleggaar and coworkers and new compounds are nowadays being reported [86,87,88,89,90,91,92]. They are meroterpenoids possessing 4 to 6 carbo and heterocycles, from which 12 of them bear an oxepane-like structure. Their basic structure is based on a pentacyclic 5/6/6/6/7 system, as shown in Figure 10. It is known that their biosynthesis has its origin in a farnesyl phthalide derivative, which, upon cyclization and oxidative modifications, furnishes this family [93,94,95]. Many beneficial effects have been found, such as anticancer, antibacterial, antiviral and antifouling properties; most of them due to specific inhibition of crucial target proteins (α-glucosidase, AP-1 transcription factor, endo-1,3-β-d-glucanase, etc.).

**Scheme 4:**
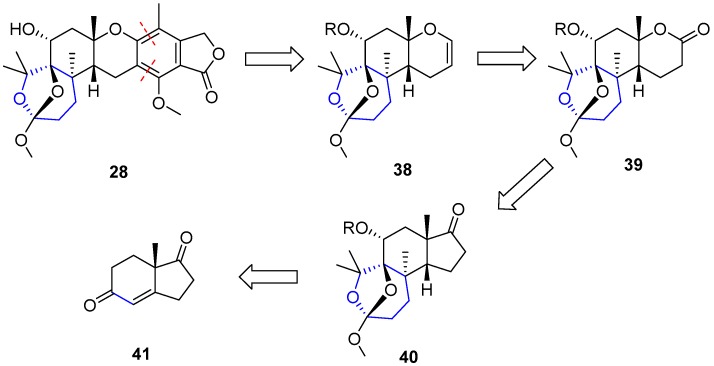
Retrosynthetic analysis towards (−)-Austalide B.

The only attempt regarding the total synthesis of a member of this family was reported by Paquette and coworkers in 1994 [96,97]. The selected molecule was (−)-Austalide B. They envisaged a retrosynthetic analysis by splitting the preparation into two sections. The so-called western sector was considered the most challenging difficulty. To access compound **38**, lactone **39** seemed to be the best precursor, which could derive from a ring expansion of a tetrahydrofuran (**40**) through a Baeyer-Villiger oxidation. Such molecule could be obtained from very simple diketone **41** after a Robinson annulation and subsequent ring expansion. On the other hand, the phthalide moiety (eastern sector) could be easily connected through a sequence of conventional proceedings to dihydropyran (**38**) (Scheme 4).

**Scheme 5:**
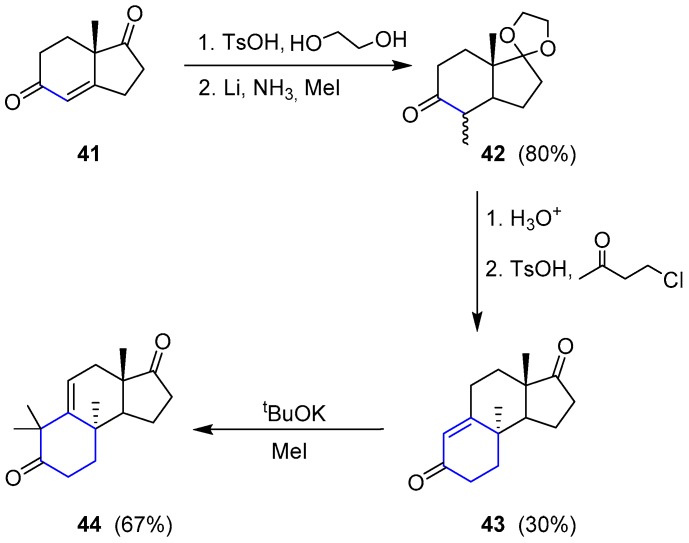
First steps towards the synthesis of the western section of (−)-Austalide B.

Starting by readily available **41**, the saturated ketone was regioselectively protected and subjected to metal reduction in the presence of methyl iodide to give **42**. Robinson annulation did not yield the expected cyclization, so the reaction with 4-chloro-2-butanone under acidic conditions was used instead to obtain **43**. This compound was dimethylated to furnish **44** and complete the first stage of the western section synthesis (Scheme 5).

**Scheme 6:**
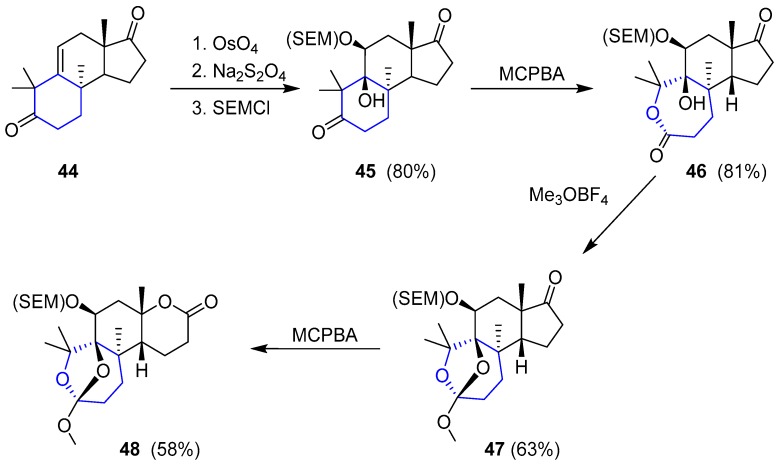
Second stage towards the synthesis of the western section of (−)-Austalide B.

Then, compound **44** was oxidized with osmium tetroxide to give a diol whose secondary alcohol was selectively protected as SEM ether **45**. Under Baeyer-Villiger conditions only the cyclohexanone moiety underwent the homologation process resulting in **46**. The second Bayer-Villiger oxidation could be accomplished upon formation of the corresponding ortho lactone **47**, thus obtaining the expected complete western section **48**. The process is summarized in Scheme 6.

**Scheme 7:**
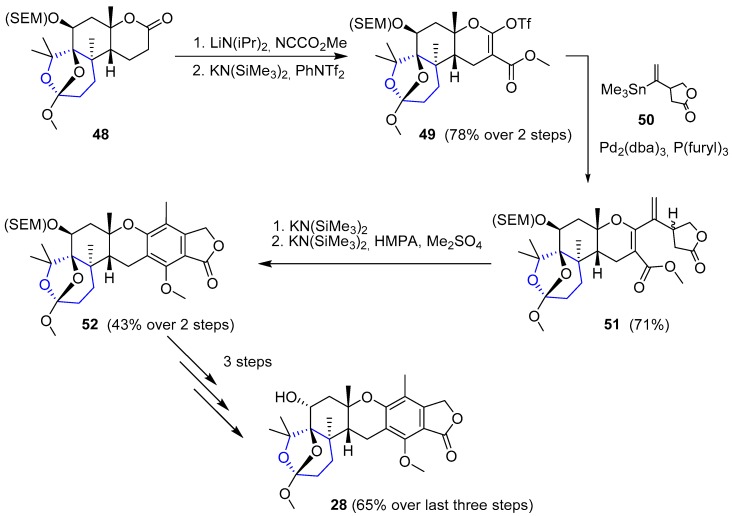
Successful eastern section construction and final obtention of (−)-Austalide B.

The synthesis of the eastern section (Scheme 7) turned out to be much harder than initially expected. Finally, after two unsuccessfully attempts, Paquette and coworkers were able to achieve desired Austalide B and the procedure is described as follows. Lactone **48** was subjected to C-alkylation (with cyanoformate) and subsequent *O*-triflation to give **49**. This triflate was coupled with previously prepared stannane **50** under standard Stille conditions to obtain compound **51**. Then, intramolecular cyclization and further benzannulation followed by methylation gave rise to **52**, whose main scaffold was very close to the natural desired product. Finally, deprotection of SEM group in the western section and inversion at C6 in a 3-step sequence ultimately yielded (−)-Austalide B (**28**).

## 6. Alkaloids

### 6.1. Bromotyrosine Alkaloids: Psammaplysins and Ceratinamides

**Figure 11:**
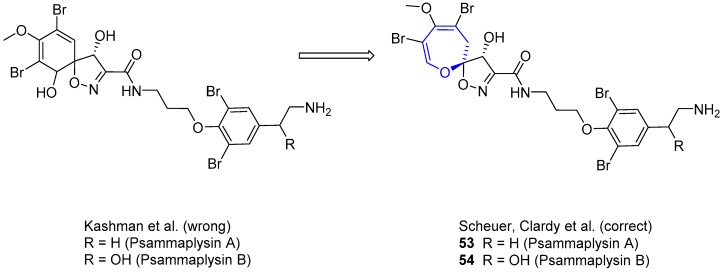
Kashman’s assigned structure of psammaplysins A and B (wrong), and later correction by Scheuer, Clardy and coworkers.

In 1983, Kashman and coworkers reported the isolation of two new compounds from sponge *Psammaplysilla purpurea* [98]. They showed antibiotic properties, being active against gram-positive bacteria and *E. coli*. Initially, their structures were wrongly assigned as spirocyclohexadienyloxazoline derivatives. Two years later, Scheuer and Clardy elucidated the correct structure (Figure 11), based on a deeper study of the NMR spectra and single-crystal X-ray diffraction [99]. The absolute configuration for psammaplysin A was assigned as (6S*,7S*), but recently it has been corrected to (6*R*,7*R*) by Garson and Kurtán [100].

**Figure 12:**
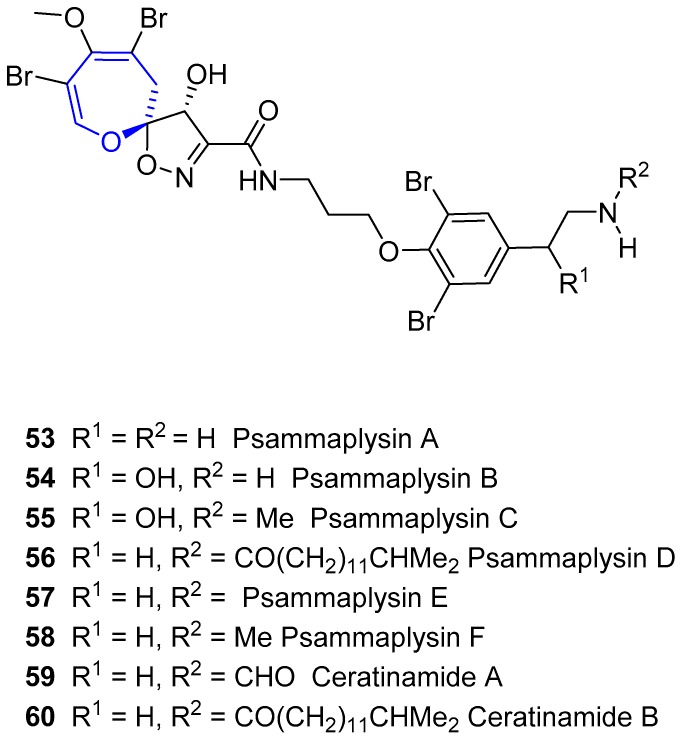
Psammaplysins A–F and Ceratinamides A and B.

In the nineties, some other members of the family were reported. Psammaplysin C was isolated from *Psammaplysilla purpurea* by Ireland and coworkers in 1992 [101]. It has identical structure to psammaplysin B, except for the amine substitution. Later on, Scheuer and coworkers reported the new psammaplysins D and E from an unknown species of *Aplysinella* sponge, collected at Pingelap Atoll, Micronesia [102]. The former has an amide group instead of the terminal amine and the latter has an unprecedented cyclopentenedione ring. In 1996, Fusetani’s group isolated ceratinamides A and B, named after the sponge they were isolated from, *Pseudoceratina purpurea* [103]. These new compounds, along with Psammaplysins A and E showed antifouling activity against *Balanus amphitrite*. Finally, psammaplysin F was reported by Schmitz and coworkers, from a sponge believed to be from the *Aplisynella* genus [104]. The structures of these compounds are shown in Figure 12.

In the 2000s no new members of this family were reported. Bewley and coworkers reported that psammaplysins A and B inhibit mycothiol-*S*-conjugate amidase from Mycobacterium tuberculosis and *Mycobacterium smegmatis*, also inhibiting growth of the latter [105,106].

In 2010, Quinn and coworkers isolated psammaplysin G from an Australian sponge of *Hyatella* species [107]. It is the first example of this family that bears a terminal *N*-methylurea moiety. Both psammaplysin F and G showed antimalarial activity. Psammaplysin G was very active against a chloroquine-resistant (Dd2) strain of *Plasmodium falciparum* at 40 µM but was not cytotoxic at all to HEK293 (a human embryonic kidney cell line). Psammaplysin F obtained better values (1.4 and 0.87 µM) against Dd2 and 3D7 (chloroquine-sensitive) strains, respectively. In 2011, the same group reported the isolation of psammaplysin H, being also active against *P. falciparum*, and quite selective (>97-fold) [108]. Ramsey and McAlpine reported antibiotic activity for psammaplysins F and H against Gram-positive bacteria. Psammaplysin F produced an unequal chromosome partitioning between daughter cells, placing it as a possible new lead antibiotic. 

**Figure 13:**
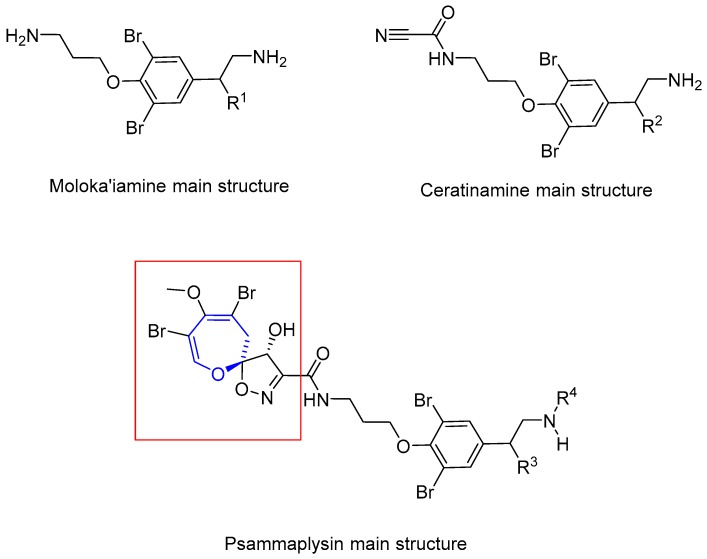
Basic structures present in moloka’iamines, ceratinamines and psammaplysins. The spirooxepinisoxazoline moiety present in the latter (red square) could be the key for their anticancer properties.

In the past few years, many other compounds have appeared. In 2012, Wright reported psammaplysins I and J from a *Suberea* species sponge [109], but did not assess their biological activity. In the same year, Garson found 21 new derivatives from the sponge *Aplysinella strongylata* [110]. Five of them had different chains attached to C-16, and the rest had terminal fatty acid chains. 19-Hydroxypsammaplysin E inhibited growth of *P. falciparum* (IC_50_ = 6.4 µM). Another four compounds of this kind were isolated by Lee and coworkers from a sponge of the genus *Suberea* in 2013 [111]. Most of them had a significant activity against several cancer cell lines. Lee also assessed some moloka’iamines and ceratinamides from the same sponge, and they exhibited no activity up to 70 µM, thus concluding that the spirooxepinisoxazoline moiety is crucial for their anticancer activity. Comparison of the structures can be seen in Figure 13.

To the best of our knowledge, no total synthesis of any of the compounds of this family has been reported.

### 6.2. Guanidinium Alkaloids

**Figure 14:**
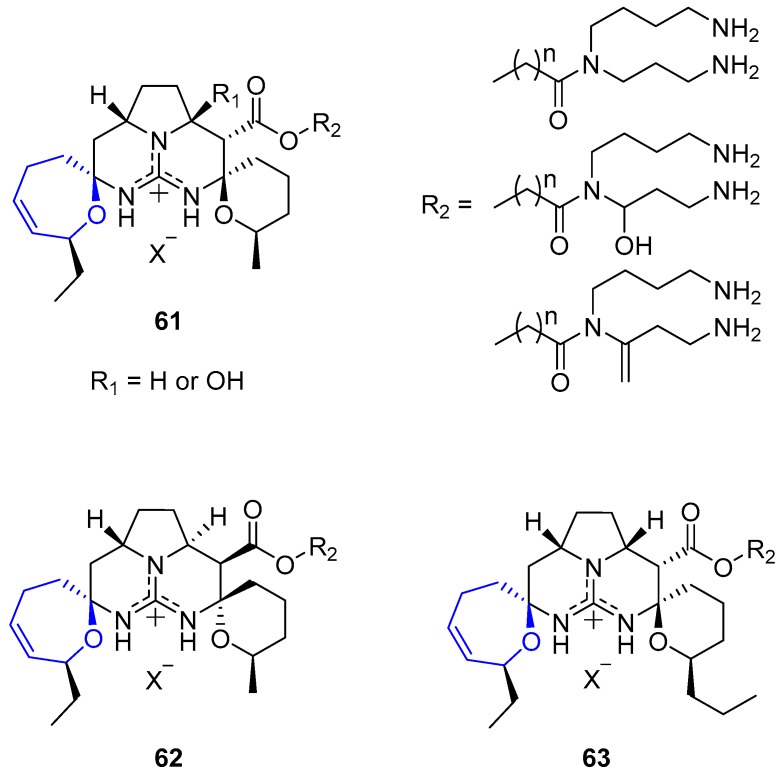
Common scaffolds for oxepane-containing guanidinium alkaloids. Vessel units (**61**, **62** and **63**); and typical anchor units (R^2^). Note that not all the structures are addressed here as R^2^ could contain other spermidine moieties or even being other functional groups (a simple alkyl chain, for instance). For a complete list of known compounds so far, see Ref. [121].

The family of guanidinium alkaloids containing oxepane rings is diverse since it contains over 15 members. Fortunately, they share some structural similarities [112]. A triazaperhydroacenaphthalene skeleton directly connected to an oxepane and a tetrahydropyran, giving rise to the so-called pentacyclic “vessel unit”, is common in all molecules. Then, a very long chain is linked to this scaffold containing a hydrocarbon fatty acid functionalized as amide with a spermidine moiety furnishing the “anchor unit”. The reason why these two parts are called like this are due to the likeness of the molecule to a macroscopic ship trailing an anchor [113]. These structures are summarized in Figure 14.

All of them have been primarily isolated from marine sponges. The first member was discovered by Kashman’s group in 1989 from *Hemimycale* sp. of the Red Sea and the Caribbean *Ptilocaulis spiculifer* [114]. Since then, a plethora of new molecules have been reported, and new names were coined, such as monanchomycalins, crambescidin, neofolitispates, etc. All of them based on the marine species from which the compound is obtained [113,114,115,116,117,118,119,120].

Given that the guanidine motif is present in arginine aminoacid and guanine nucleobase, it is not surprising that this functional group appears in countless natural products, and it is even less surprising that it possesses outstanding beneficial properties. It has been demonstrated that this family show antiviral [122], fungicidal [123] and anticancer effects [124,125,126,127,128]. The mechanisms of action for this latter activity has been studied and now it is known that they are excellent Ca^2+^-channel blockers and good inhibitors of enzymes Na^+^, K^+^ or Ca^2+^-ATP-ase [129,130]. There are other applications; for instance, Crambescidin 800 has revealed exceptional protection of cell lines under oxidative stress, typically present in neuronal degenerative diseases [131].

Concerning the synthesis of the members in this family, fantastic efforts towards the development of chemical tools to prepare these intriguing structures have been addressed [121,132,133,134,135,136,137,138,139,140]. Since there are several in-depth works regarding the total synthesis of many compounds (or their fragments) in the family [141,142,143,144,145], addressing all of them would be out of the scope of this review. Therefore, we will cover herein, as an example, the preparation of the simple Crambescidin 359 which structure coincides with the main core “vessel unit”.

**Scheme 8:**
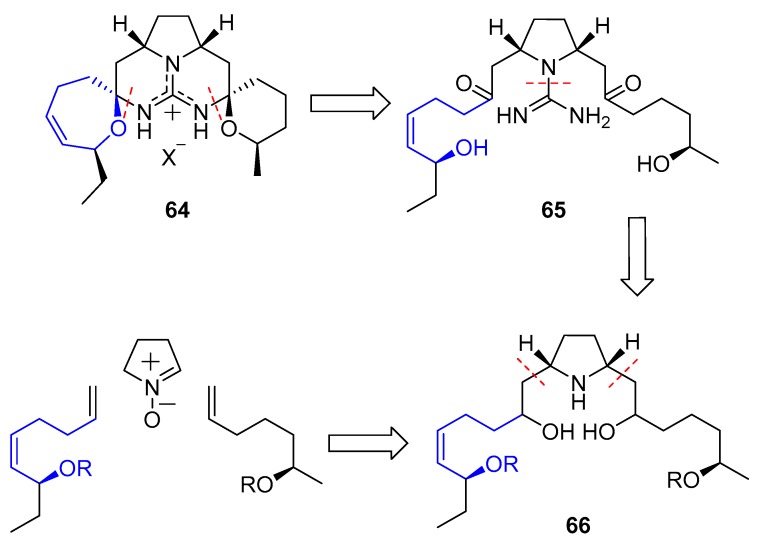
Retrosynthetic analysis towards Crambescidin 359 through a double 1,3-dipolar cycloaddition between a nitrone and two terminal olefins.

In 2002, Nagasawa and coworkers published the first total synthesis of this natural product [146]. They conceived a double 1,3-dipolar cycloaddition between a commercially available nitrone and two distinct terminal olefins which would have been previously prepared. Resulting pyrrolidine **66** would easily yield guanidine **65** and, finally, a double intramolecular condensation would furnish the desired compound (**64**), as shown in Scheme 8.

**Scheme 9:**
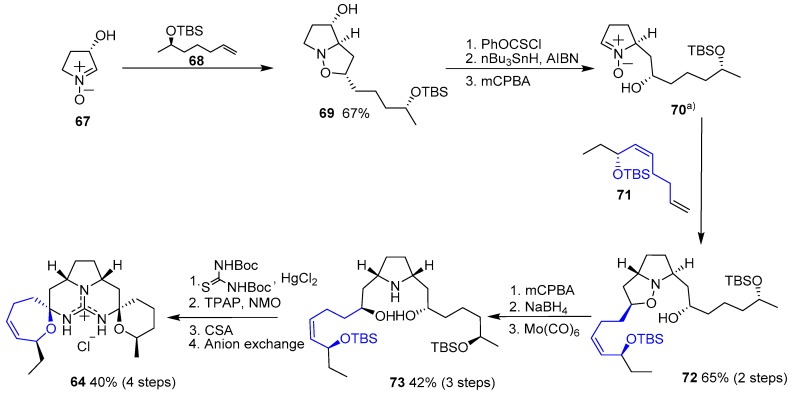
Summarized synthesis of Crambescidin 359 by Nagasawa’s group. ^a)^ Compound **70** was directly used for the next reaction and yield is not reported.

1,3-Dipolar cycloaddition of nitrone **67** with alkene **68** yielded isoxazolidine **69**. Then, removal of the hydroxyl group, followed by treatment with mCPBA provided free nitrone **70**. A second cycloaddition with olefin **71** furnished compound **72**, which was readily transformed in pyrrolidine **73** by oxidation with mCPBA and subsequent reduction. Reaction with bis-*N*-Boc thiourea, followed by oxidation with TPAP/NMO system, deprotection with mild camphorsulfonic acid (CSA) and final *N*,*O*-acetalization gave **64** (Scheme 9).

**Scheme 10:**
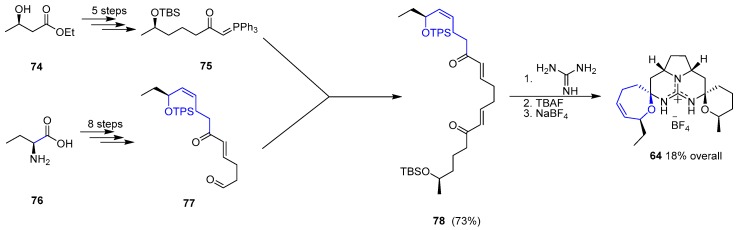
Alternative synthesis developed by Murphy towards Crambescidin 359.

One year later Murphy and coworkers reported an alternative synthetic approach to **64** [147]. The strategy relied on the preparation of a suitable bis-enone **78** ready to couple with a guanidine moiety to provide the pentacyclic unit of **64** in a one-step process (Scheme 10). Hence, a convergent synthesis of two different blocks (**75** and **77**) gave access to desired bis-enone **78** in good overall yield (14 steps). Condensation with guanidine followed by deprotection of two silyl groups (TBS and TPS) furnished Crambescidin 359 (**64**) bearing BF_4_ anion upon treatment with NaBF_4_.

**Scheme 11:**
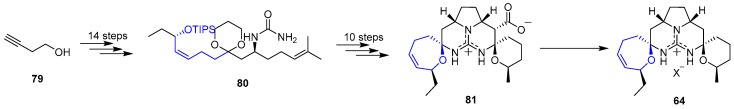
Overman’s synthesis of Crambescidin 359 upon decarboxylation of precursor **81**.

On the course of synthetic studies towards guanidinium alkaloids, Overman’s group published a different stepwise method [148] that started with commercially available 3-butynol (**79**) whose chain was elongated with the incorporation of a guanidine group to get **80**. Then, a set of 10 steps allowed the synthesis of guanidinium carboxylate **81**. This compound was an extraordinary precursor for the preparation of other crambescidinds (and more members in guanidinium alkaloids family) and, due to that reason, it was extensively studied. Interestingly, it was found that, upon standing **81** in several buffers, decarboxylation mildly occurred yielding Crambescidin 359 (**64**). This approach is summarized in Scheme 11.

### 6.3. Oxepinamides Family

This large group has been isolated from a set of marine fungus species associated to very distinct organisms. They possess interesting beneficial properties; however, they are especially important due to their anti-cancer activity [149]. They all bear an oxepin fused to a pyrimidinone moiety, as the main structure, and a six- or seven-membered lactam. In the first case, the compounds are also known as diketopiperazines, which correspond to another vast family of species (marine or not), for which oxepane-derived molecules will be addressed later on.

**Figure 15:**
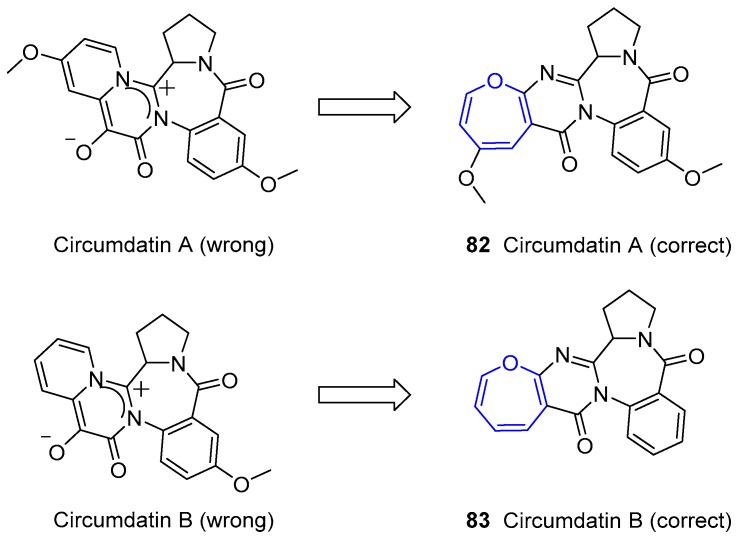
Circumdatins A and B. Original zwitterionic structure proposals by Christophersen (**Left**), and revised definitive structures by Kusumi (**Right**).

The first example of this family, called Cinereanin, was isolated by Springer, Arison, Roberts and collaborators from *Botrytis cinereal* sunflower seeds in 1988 and showed to have plant growth regulating properties [150]. Its structure remained unknown for years until Christophersen’s group reported the isolation of three benzodiazepine alkaloids (called Circumdatins A, B and C) from a culture of the fungus *Aspergillus ochraceus* [151]. Although it is known that this is a common soil fungus, it has been demonstrated its adaptation to other niches, such as marine ecosystem. Interestingly, the initial zwitterionic proposed structures of two of them (A and B) were wrongly assigned according to recorded NMR data. In fact, no oxepane-like group was present. This misled the scientific community for nine years until Kusumi and coworkers could isolate a set of alkaloids from fungus *Aspergillus ostianus* and grow single-crystals of Circumdatins A and B suitable for X-ray analysis [152], which yielded the final corrected structure as shown in Figure 15. These compounds were also detected and confirmed by Alfonso, Botana and collaborators from original fungus *Aspergillus ochraceus* [153].

**Figure 16:**
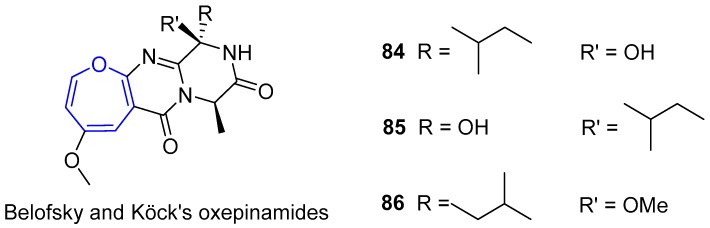
Chemical structures of Oxepinamides A–C isolated by Belofsky and Köck.

In parallel to Circumdatins discoveries, Belofsky, Köck’s group was able to isolate several bioactive metabolites from a fungus of the genus *Acremonium*, which were collected from the surface of Caribbean tunicate *Ecteinascidia turbinata* [154]. Three of them, generally called Oxepinamides A–C, contained oxepin derivatives with a structure very similar to the first one described by Springer, Arison, Roberts and collaborators, as depicted in Figure 16. It is worth noting that Oxepinamide A was capable of inhibiting ear edema induced in mice by using resiniferatoxin.

**Figure 17:**
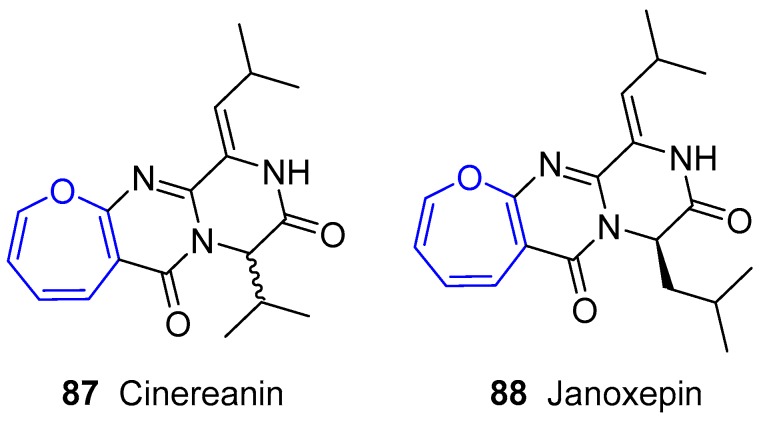
Comparison of Cinereanin (**Left**) and Janoxepin (**Right**) structures.

Later on, Sprogøe’s group reported two new compounds extracted from fungus *Aspergillus janus* [155]. One of them was called Janoxepin and had a very close structure to Cinereanin (Figure 17). It turned out to be active against the malaria parasite *Plasmodium falciparum* 3D7.

**Scheme 12:**
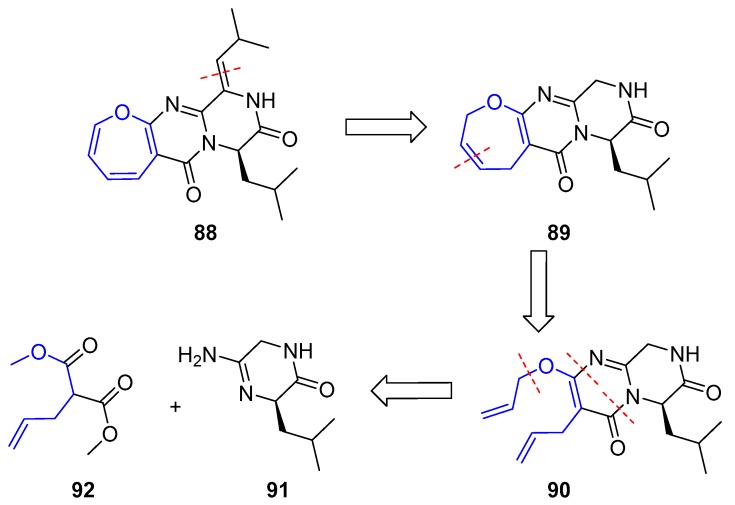
Retrosynthetic analysis of Janoxepin.

Janoxepin is the only compound of this family of oxepinamides from fungus species that has been synthesized. In 2012 Taylor’s group reported its total synthesis [156], according to the following retrosynthetic analysis: the key step is a ring closing metathesis (RCM) of a diallylated pyrimidinone **90**, which should be easily prepared from guanidine **91** and dimethyl allylmalonate (Scheme 12).

**Scheme 13:**
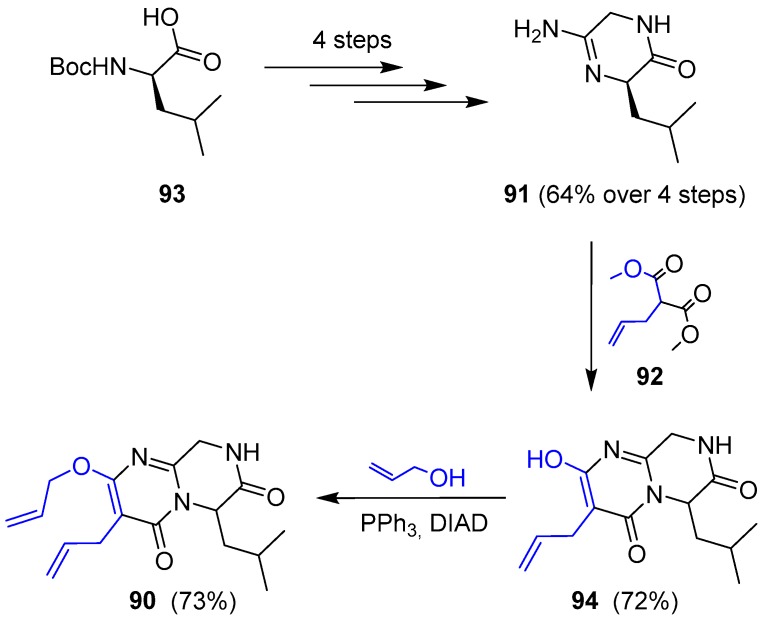
Main sequence towards diallylated pyrimidone **90** starting from *N*-Boc-d-leucine **93**.

Thus, amidine **91** was obtained in good yield in four consecutive steps (coupling with aminoacetonitrile, Boc deprotection, oxime formation-hydrogenation and final cyclization) starting from commercially available *N*-Boc-d-leucine. Further condensation with malonate **92** gave pyrimidone **94** along with unwanted total racemization (only 5% ee was detected). This molecule was turned into compound **90** by Mitsunobu reaction with allylic alcohol (Scheme 13).

**Scheme 14:**
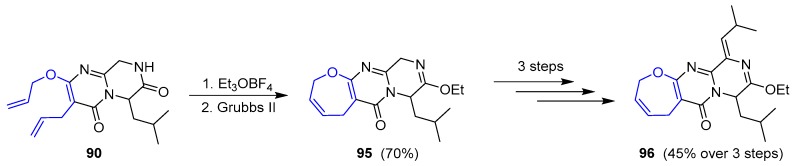
Synthesis sequence towards dihydro-oxepin and side chain incorporation.

Olefin metathesis was readily performed using second-generation Grubb’s catalyst, prior protection of **90** as imidate **95**. Resulting compound **95** underwent the incorporation of the side chain through an aldol reaction with *iso*-butyraldehyde, followed by a chlorination and dehydrochlorination sequence furnishing dihydro-oxepin **96** (Scheme 14).

**Scheme 15:**
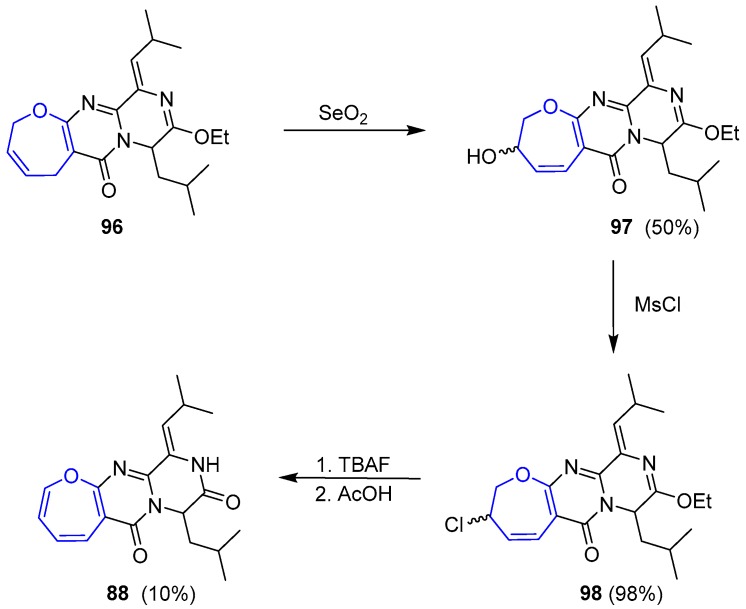
Final steps towards (±)-Janoxepin (**88**).

Formation of desired oxepin turned out to be extremely troublesome and, after many attempts, the only feasible procedure consisted of an allylic oxidation with SeO_2_ to give an alcohol **97** that was readily substituted by a chloride **98**. Dehydrohalogenation was achieved but yields could never be optimized and only 10% of **98** was obtained. Nevertheless, (±)-Janoxepin (**88**) could be finally prepared after a simple deprotection step (Scheme 15).

**Figure 18:**
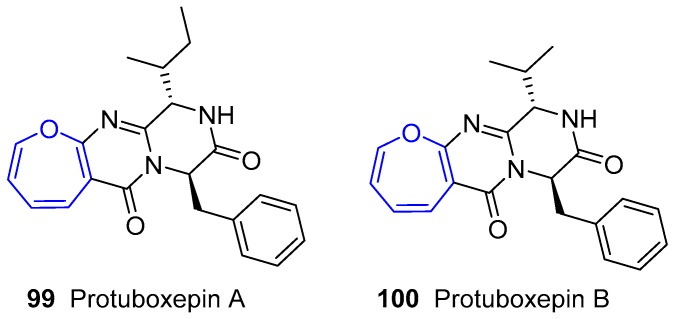
Protuboxepins A and B from marine fungus *Aspergillus* sp. SF-5044.

Ahn, Oh and collaborators described, in 2011, four compounds from an ethyl acetate fraction of the marine-derived fungus *Aspergillus* sp. SF-5044 collected from Dadaepo Beach, Busan, Korea [157]. Two of them were oxepinamides, called Protuboxepins A and B, whose structure is shown in Figure 18. The reader should rapidly realize that these piperazines are structurally similar to Cinereanin and Janoxepin. Interestingly, both compounds contain the rare d-phenylalanine amino acid fragment. However, their anti-cancer activities towards several carcinoma cell lines were moderate. Nonetheless, Kihm and Ahn’s group reported the mechanism of tumor cell growth inhibition by Protuboxepin A [158]. They demonstrated that, due to an α,β-tubulin binding, microtubule dynamics were modified in such an extent that chromosomes were misaligned in metaphase, finally producing cell apoptosis.

**Figure 19:**
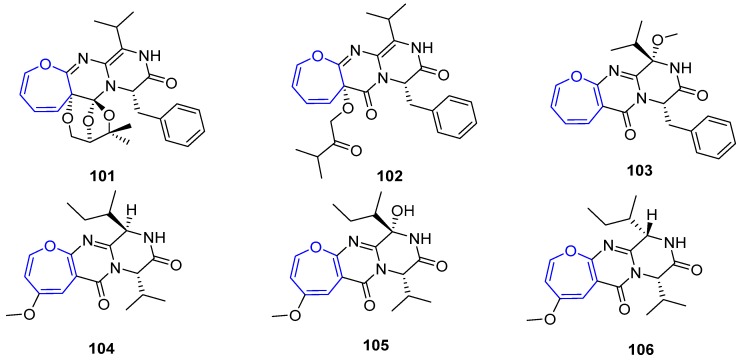
Latest oxepinamides. (**101**) Varioxepin A. (**102**) and (**103**) oxepinamides from *Aecilomyces variotii* EN-291. (**104**) and (**105**) oxepinamides from *Aspergillus versicolor* SCSIO 05879. (**106**) oxepinamides from vent-crab associated *Aspergillus versicolor* XZ-4.

In the last few years, up to six new rare oxepinamides have emerged from different fungus species [159,160,161,162]. They resemble structures already described here and are depicted in Figure 19. Apart from their structural interest, they have showed new bioactive abilities, such as antibacterial and antifungal properties.

### 6.4. Diketopiperazine Sulfides

As commented above, diketopiperazine structures bearing oxepane-like moieties are present in other families. One interesting family corresponds to disulfide (trisulfide or tetrasulfide) derivatives. Due to the presence of such functional group, all these compounds exhibit outstanding biomedical applications (anticancer, antifungal, antiviral, etc.) [163,164,165,166,167,168,169,170,171].

**Figure 20:**
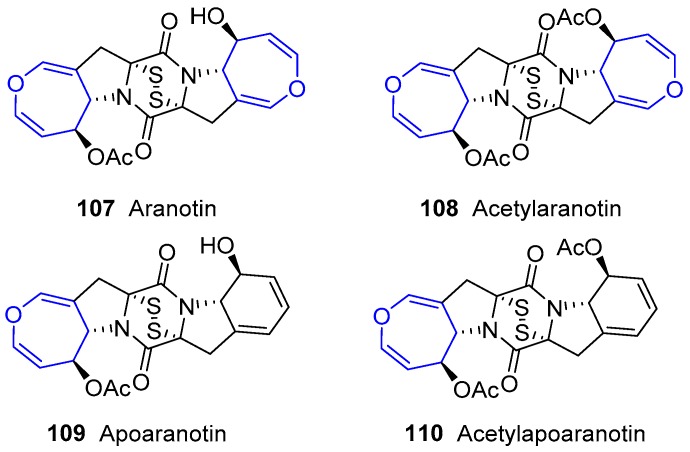
First set of oxepin-containing diketopiperazine disulfides isolated from fungus *Arachniotus aureus*.

The first members in this family were obtained from fungus *Arachniotus aureus* in 1968, and were named Aranotin and Apoaranotin. Along with their acetylated derivatives, they were considered metabolites from such species and very related to formerly known sulfur-containing diketopiperazines, e.g., Gliotoxin and Sporidesmin (Figure 20) [172,173,174,175,176]. Structural characteristics are a central diketopiperazine scaffold, a disulfur bridge and a functionalized oxepin-fused pyrrolidine. Their biosynthesis pathway has been recently unraveled by Wang and collaborators using genome-based deletion analysis [177]. These compounds are present in other classes of fungus, such as *Aspergillus* sp. KMD 901, and showed excellent apoptosis-inducing effects towards colon cancer cell lines [178].

**Figure 21:**
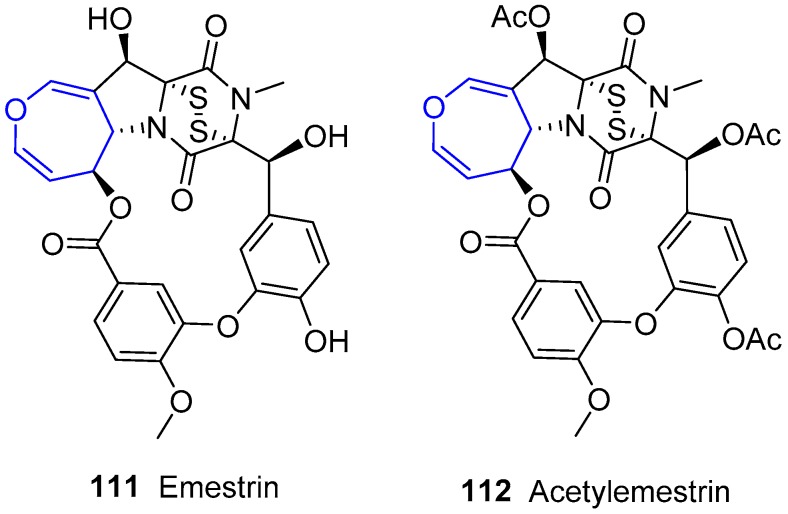
Emestrin and its acetylated derivative isolated from *Emericella striata*.

Another molecule of this family was obtained by Kawai’s group from *Emericella striata*, a thermotolerant fungus collected in Nepal [179,180,181]. It was called Emestrin and was reported as its acetylated form (Figure 21). Although not strictly marine, this compound belongs to the family due to its similarity to the previous structures and it is worth addressing. The previously described main scaffold is maintained but, in this case, diketopiperazine and oxepin moieties are additionally connected through a diphenyl ether chain giving rise to a macrocyclic lactone. Years later, the same group found structurally related molecules from *Emericella foveolata* [182].

**Figure 22:**
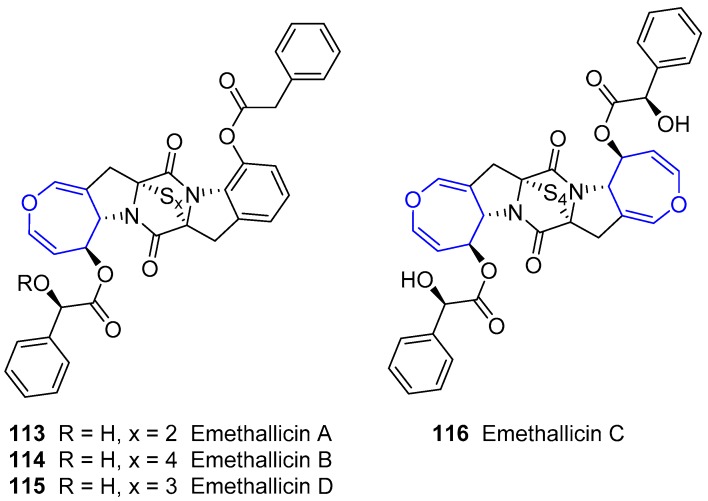
Structures of oxepin-containing emethallicins, their acetylated forms being R = OAc.

The same researchers reported the isolation of a small group of diketopiperazines arising from *Emericella heterothallica* extracts which were called Emethallicins [183,184]. Their structures resemble those from Aranotin, having a different acetylating group and a variable number of sulfur atoms (Figure 22). This feature was also observed for already described Emestrin derivatives. They have shown antiallergic properties owing to the observation of histamine release inhibition from mast cells and towards 5-lipoxygenase.

**Scheme 16:**
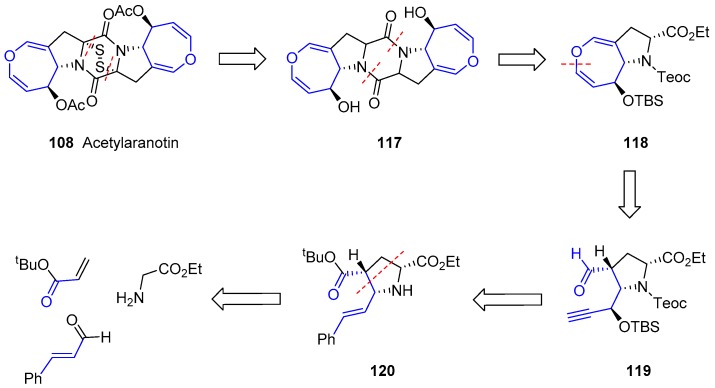
Retrosynthetic analysis of (−)-Acetylaranotin.

Interestingly, the synthesis of the intriguing structures of this family has attracted the attention of several authors. Thus, many synthetic approaches towards the partial synthesis of these compounds or analogues have been carried out and published [185,186,187,188,189,190,191,192]. In 2012, Reisman and coworkers reported the first total synthesis of an oxepin-containing diketopiperazine disulfide, (−)-Acetylaranotin, over 40 years after its discovery [193]. Due to the C_2_-symmetric nature of this compound, the strategy to synthesize this compound was based on the obtention of an oxepin fused to proline **118** which, after dimerization product **117** and a final step of sulfenylation, could provide (−)-Acetylaranotin. Proline oxepin **118** was foreseen to be prepared by a metal vinylidene-mediated 7-*endo* cycloisomerization from an alkynal precursor (**119**) that could be obtained from proline **120**. This molecule can be synthesized through an asymmetric 1,3-dipolar cycloaddition of an acrylate and the azomethine ylide resulting from the coupling of a glycinate and cinnamaldehyde (Scheme 16).

**Scheme 17:**
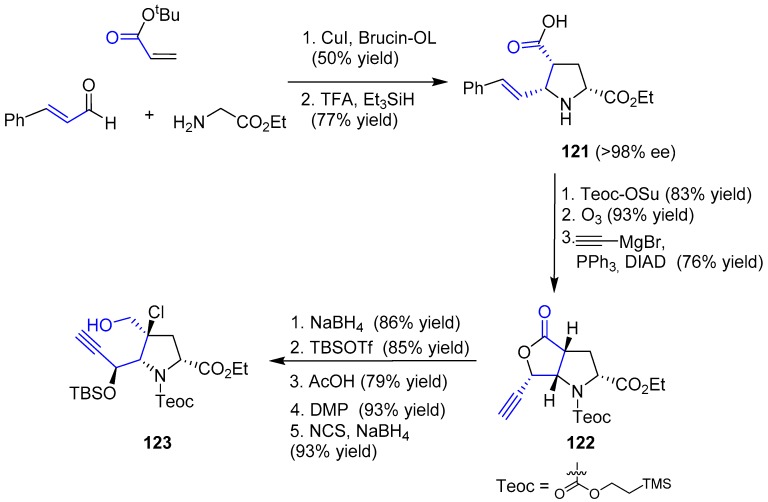
First stage of Acetylaranotin preparation. Synthetic sequence towards alkynol proline **123**.

The enantioselective synthesis of pyrrolidine **121** was achieved by an asymmetric 1,3-dipolar cycloaddition mediated by catalytic copper iodide and Brucin-OL as the chiral ligand, followed by hydrosilane-reduction and deprotection of *t*Bu group. Then, four consecutive steps, such as Teoc-protection, ozonolysis, addition of ethynyl Grignard reagent and final Mitsunobu reaction, gave rise to chiral alkynyl lactone **122**. Finally, reduction with sodium borohydride, protection of the resulting alcohol and a sequence of Dess-Martin oxidation, chlorination of the α carbon and aldehyde reduction furnished the desired alkynol **123**. The whole first stage is summarized in Scheme 17.

**Scheme 18:**
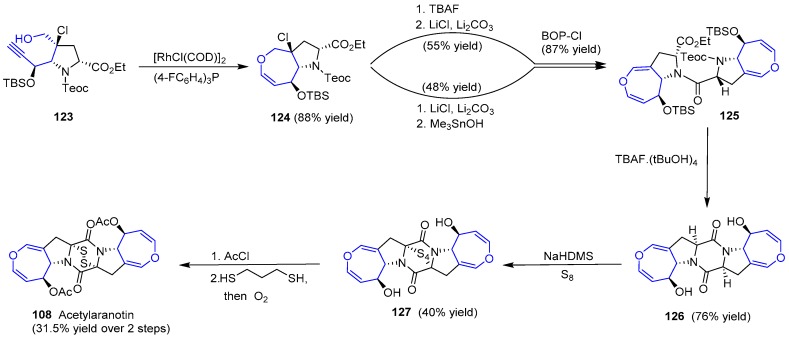
Second stage of Acetylaranotin preparation.

The second stage consisted of the construction of oxepin structure, dimerization and final sulfenylation. The use of catalytic [RhCl(COD)]_2_ in the presence of tris(*p*-fluorophenyl)phosphine yielded the expected oxepin **124**, which was subjected to two different procedures. In both, a step of dehydrohalogenation and deprotection of orthogonal functional groups (carbamate into amine and ester into acid) resulted in two complementary peptides that reacted in the presence of BOP-Cl, a common reagent to activate carboxyl groups for peptide coupling, to get **125**. Treatment with TBAF allowed the second amide formation (**126**). Surprisingly, epimerization of both stereogenic carbons in diketopiperazine skeleton occurred, but the mechanism was unclear. Nevertheless, episulfide formation was the last difficulty to be overcome. Treatment with NaHDMS and S_8_ furnished a tetrasulfide (**127**) with the appropriate stereochemistry which, after diacetylation of two hydroxyl groups, reduction with propanedithiol and further mild oxidation of the resulting dithiol gave expected (−)-Acetylaranotin (Scheme 18). The whole process required 18 steps and the key step relied on a Rh-catalyzed 7-*endo* cyclization to obtain the monomeric oxepin **124**.

**Scheme 19:**
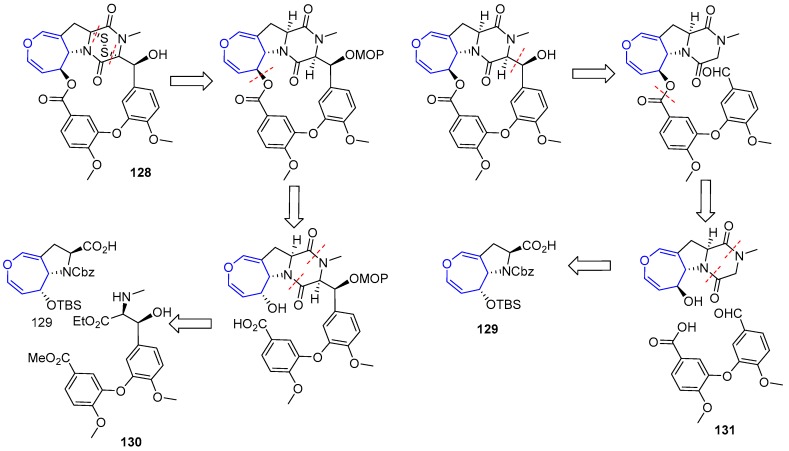
Retrosynthetic analyses for the formation of MPC-1001B. Note that compound **129** is considered a key intermediate present in both schemes. Anisic acid derivatives **130** and **131** were easily prepared.

In recent years, other diketopiperazin sulfides total syntheses have been disclosed based on the contribution made by Reisman [193]. Compound (+)-MPC1001B (**128**), a molecule closely related to Emestrin, was prepared in 2004 [194,195]. The key step of the process involved the elusive 15-membered macrolactone formation and two plausible retrosynthetic analyses were devised. In both, sulfenylation would take place in the final step and oxepin proline moiety (**129**) was considered as the key intermediate from which macrocycle would be built. The main difference relied on the way the macroring would be closed. In the first one, a Mitsunobu reaction through an alcohol directly linked to oxepin fragment is proposed, whereas in the second one an aldol reaction in diketopiperazin moiety would be carried out (Scheme 19).

**Scheme 20:**
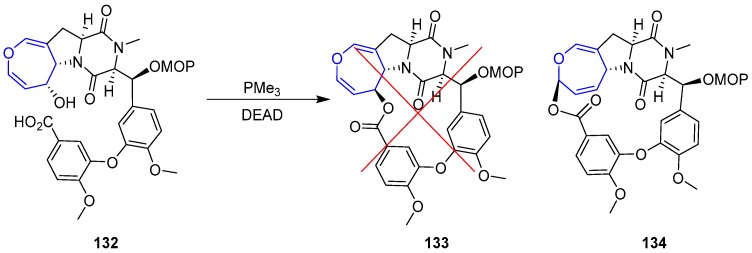
First unfruitful approach towards 15-membered macrolactone **133**.

Unfortunately, macrocyclization of **132** under Mitsunobu conditions failed to provide the desired lactone **133**, instead regioisomer **134** was isolated in high yields (Scheme 20). 

**Scheme 21:**
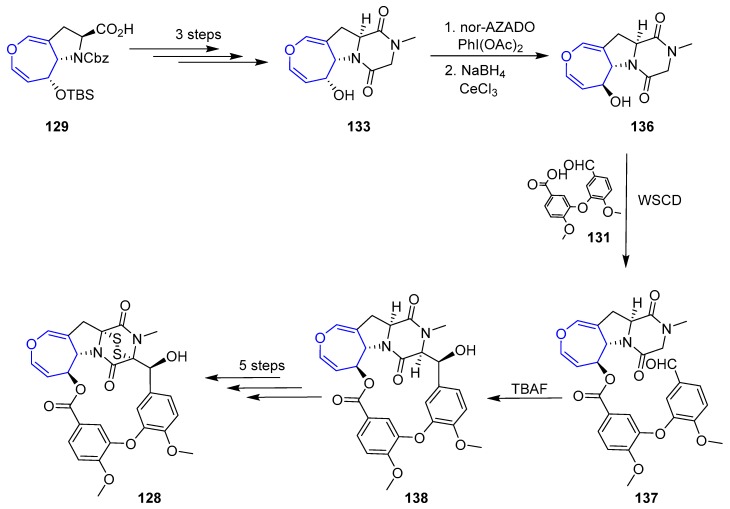
Total synthesis of (+)-MPC10001B (**128**).

From easily accessible proline **129**, a key precursor of the second strategy, was readily obtained diketopiperazine **133**. Inversion of the configuration at C-6 (via a mild oxidation and Luche reduction), followed by condensation with anisic acid derivative **131** using WSCD provided ester **137**. To the delight of the authors, now the crucial formation of the macrolactone (**138**) formation could be easily achieved in the presence of TBAF. From that point, sulfenylation was carried out using a modification of Reisman’s protocol (with a novel trityl trisulfide reagent) although the secondary alcohol present in the structure had to be protected in the process, increasing the expected number of steps. Nevertheless, elusive diketopiperazin disulfide (+)-MPC1001B could be obtained for the first time (Scheme 21).

Simultaneously, Reisman’s group also reported the total synthesis of (−)-Acetylapoaranotin using a very close methodology [196]. Owing to the nonsymmetric nature of this compound, the procedure had to be a step-wise preparation of two proline key intermediates which were readily coupled and finally sulfenylated.

In summary, marine drugs containing 7-membered oxacycles are abundant in nature. They show a rich variety of structures whose intriguing features have attracted the attention of many synthetic chemists. Moreover, their relevant biological properties include antimalarial, antifungal, antibacterial activities and, most remarkably, high cytotoxicity against a wide number of cancer cells. In this review, we have tried to show an overview of these types of compounds, focusing on their isolation, structure determination, biological properties and synthetic approaches.

## References

[1] Blunt J.W., Copp B.R., Hu W.-P., Munro M.H.G., Northcote P.T., Prinsep M.R. (2008). Marine natural products. Nat. Prod. Rep..

[2] Fujiwara K., Kiyota H. (2006). Total Synthesis of Medium-Ring Ethers from Laurencia Red Algae. Marine Natural Products.

[3] Nicolaou K.C., Frederick M.O., Aversa R.J. (2008). The Continuing Saga of the Marine Polyether Biotoxins. Angew. Chem. Int. Ed..

[4] Vilotijevic I., Jamison F.T. (2010). Synthesis of Marine Polycyclic Polyethers via Endo-Selective Epoxide-Opening Cascades. Mar. Drugs.

[5] Smith E.T. (2017). Biogenetic Relationships of Bioactive Sponge Merotriterpenoids. Mar. Drugs.

[6] Fernandez J.J., Souto M.L., Norte M. (2000). Marine polyether triterpenes. Nat. Prod. Rep..

[7] Faulkner D.J. (1994). Marine natural products. Nat. Prod. Rep..

[8] Pettit G.R., Herald C.L., Allen M.S., Von Dreele R.B., Vanell L.D., Kao J.P.Y., Blake W. (1977). Antineoplastic agents. 48. The isolation and structure of aplysistatin. J. Am. Chem. Soc..

[9] Palaniveloo K., Vairappan C.S. (2014). Chemical relationship between red algae genus Laurencia and sea hare (Aplysia dactylomela Rang) in the North Borneo Island. J. Appl. Phycol..

[10] De Nys R., Steinberg P.D., Rogers C.N., Charlton T.S., Duncan M.W. (1996). Quantitative variation of secondary metabolites in the sea hare Aplysia parvula and its host plant, Delisea pulchra. Mar. Ecol. Prog. Ser..

[11] Rogers C.N., De Nys R., Charlton T.S., Steinberg P.D. (2000). Dynamics of Algal Secondary Metabolites in Two Species of Sea Hare. J. Chem. Ecol..

[12] Pennings S.C., Paul V.J. (1993). Sequestration of dietary secondary metabolites by three species of sea hares: Location, specificity and dynamics. Mar. Biol..

[13] Paul V.J., Fenical W. (1980). Palisadins A, B and related monocyclofarnesol-derived sesquiterpenoids from the red marine alga *Laurencia* cf. palisada. Tetrahedron Lett..

[14] Capon R., Ghisalberti E.L., Jefferies P.R., Skelton B.W., White A.H. (1981). Sesquiterpene metabolites from laurencia filiformis. Tetrahedron.

[15] Wright A.D., König G.M., Sticher O. (1991). New Sesquiterpenes and C15 Acetogenins from the Marine Red Alga Laurencia implicata. J. Nat. Prod..

[16] De Nys R., Wright A.D., König G.M., Sticher O., Alino P.M. (1993). Five New Sesquiterpenes from the Red Alga Laurencia flexilis. J. Nat. Prod..

[17] Su J.-Y., Zhong Y.-L., Zeng L.-M., Wu H.-M., Ma K. (1995). Terpenoids from Laurencia karlae. Phytochemistry.

[18] Kuniyoshi M., Marma M.S., Higa T., Bernardinelli G., Jefford C.W. (2001). New Bromoterpenes from the Red Alga Laurencia luzonensis. J. Nat. Prod..

[19] Su H., Yuan Z.-H., Li J., Guo S.-J., Deng L.-P., Han L.-J., Zhu X.-B., Shi D.-Y. (2009). Sesquiterpenes from the Marine Red Alga Laurencia saitoi. Helv. Chim. Acta.

[20] Su H., Shi D.-Y., Li J., Guo S.-J., Li L.-L., Yuan Z.-H., Zhu X.-B. (2009). Sesquiterpenes from Laurencia similis. Molecules.

[21] Lee Tan K., Matsunaga S., Vairappan C.S. (2011). Halogenated chamigranes of red alga Laurencia snackeyi (Weber-van Bosse) Masuda from Sulu-Sulawesi Sea. Biochem. Syst. Ecol..

[22] Von Dreele R.B., Kao J.P.Y. (1980). The structure of the sesquiterpene aplysistatin. Acta Crystallogr. B.

[23] König G.M., Wright A.D., Sticher O., Angerhofer C.K., Pezzuto J.M. (1994). Biological Activities of Selected Marine Natural Products. Planta Med..

[24] Vairappan C.S., Kamada T., Lee W.-W., Jeon Y.-J. (2013). Anti-inflammatory activity of halogenated secondary metabolites of Laurencia snackeyi (Weber-van Bosse) Masuda in LPS-stimulated RAW 264.7 macrophages. J. Appl. Phycol..

[25] Wijesinghe W.A., Kim E.A., Kang M.C., Lee W.W., Lee H.S., Vairappan C.S., Jeon Y.J. (2014). Assessment of anti-inflammatory effect of 5beta-hydroxypalisadin B isolated from red seaweed Laurencia snackeyi in zebrafish embryo in vivo model. Environ. Toxicol. Pharmacol..

[26] Hoye T.R., Kurth M.J. (1979). Total synthesis of dl-aplysistatin. J. Am. Chem. Soc..

[27] Hoye T.R., Caruso A.J., Dellaria J.F., Kurth M.J. (1982). Two syntheses of dl-aplysistatin. J. Am. Chem. Soc..

[28] Shieh H.-M., Prestwich G.D. (1982). Chiral, biomimetic total synthesis of (−)-aplysistatin. Tetrahedron Lett..

[29] White J.D., Nishiguchi T., Skeean R.W. (1982). Stereoselective, biogenetically patterned synthesis of (.+-.)-aplysistatin. J. Am. Chem. Soc..

[30] Gosselin P., Rouessac F. (1983). Polycyclisations cationiques de polyenes via leurs bromohydrines—II synthese de la (±) aplysistatine. Tetrahedron Lett..

[31] Kraus G.A., Gottschalk P. (1983). A direct synthesis of .beta.-hydroxybutyrolactones. Total synthesis of dendrolasin and formal total synthesis of aplysistatin. J. Org. Chem..

[32] Tanaka A., Otsuka S., Yamashita K. (1984). Biomimetic Total Synthesis of (−)-Aplysistatin. Agric. Biol. Chem..

[33] Tanaka A., Suzuki M., Yamashita K. (1986). Total Synthesis of (+)-Palisadin A and (+)-12-Hydroxypalisadin B. Agric. Biol. Chem..

[34] Couladouros E.A., Vidali V.P. (2004). Novel Stereocontrolled Approach to syn- and anti-Oxepene-Cyclogeranyl trans-Fused Polycyclic Systems: Asymmetric Total Synthesis of (−)-Aplysistatin, (+)-Palisadin A, (+)-Palisadin B, (+)-12-Hydroxy-Palisadin B, and the AB Ring System of Adociasulfate-2 and Toxicol A. Chem. Eur. J..

[35] Dunlop R., Wells R. (1979). Isolation of Some Novel Diterpenes from a Soft Coral of the Genus Lobophytum. Aust. J. Chem..

[36] Raju B.L., Subbaraju G.V., Rao C.B., Trimurtulu G. (1993). Two New Oxygenated Lobanes from a Soft Coral of Lobophytum Species of the Andaman and Nicobar Coasts. J. Nat. Prod..

[37] Shin J., Fenical W. (1991). Fuscosides A-D: Anti-inflammatory diterpenoid glycosides of new structural classes from the caribbean gorgonian Eunicea fusca. J. Org. Chem..

[38] Marchbank D.H., Berrue F., Kerr R.G. (2012). Eunicidiol, an Anti-inflammatory Dilophol Diterpene from Eunicea fusca. J. Nat. Prod..

[39] Hamada T., Kusumi T., Ishitsuka M.O., Kakisawa H. (1992). Structures and Absolute Configuration of New Lobane Diterpenoids from the Okinawan Soft Coral Sinularia flexibilis. Chem. Lett..

[40] Kusumi T., Hamada T., Ishitsuka M.O., Ohtani I., Kakisawa H. (1992). Elucidation of the relative and absolute stereochemistry of lobatriene, a marine diterpene, by a modified Mosher method. J. Org. Chem..

[41] Chai M.-C., Wang S.-K., Dai C.-F., Duh C.-Y. (2000). A Cytotoxic Lobane Diterpene from the Formosan Soft Coral Sinularia inelegans. J. Nat. Prod..

[42] Bonnard I., Jhaumeer-Laulloo S.B., Bontemps N., Banaigs B., Aknin M. (2010). New Lobane and Cembrane Diterpenes from Two Comorian Soft Corals. Mar. Drugs.

[43] Edrada R.A., Proksch P., Wray V., Witte L., van Ofwegen L. (1998). Four New Bioactive Lobane Diterpenes of the Soft Coral Lobophytum pauciflorum from Mindoro, Philippines. J. Nat. Prod..

[44] Kosugi H., Yamabe O., Kato M. (1998). Synthetic study of marine lobane diterpenes: Efficient synthesis of (+)-fuscol. J. Chem. Soc. Perkin Trans. 1.

[45] Kato M., Kosugi H., Ichiyanagi T., Yamabe O. (1999). Synthetic study of marine lobane diterpenes. Enantioselective syntheses of lobatrienolide and lobatrientriol from (+)-nopinone. J. Chem. Soc. Perkin Trans. 1.

[46] Marchbank D.H., Kerr R.G. (2011). Semisynthesis of fuscoside B analogues and eunicosides, and analysis of anti-inflammatory activity. Tetrahedron.

[47] Edrada R.A., Wray V., Handayani D., Schupp P., Balbin-Oliveros M., Proksch P. (2000). Structure-activity relationships of bioactive metabolites from some Indo-Pacific marine invertebrates. Stud. Nat. Prod. Chem..

[48] Menna M., Imperatore C., D’Aniello F., Aiello A. (2013). Meroterpenes from Marine Invertebrates: Structures, Occurrence, and Ecological Implications. Mar. Drugs.

[49] Hegazy M., Mohamed T., Alhammady M., Shaheen A., Reda E., Elshamy A., Aziz M., Paré P. (2015). Molecular Architecture and Biomedical Leads of Terpenes from Red Sea Marine Invertebrates. Mar. Drugs.

[50] Shmueli U., Carmely S., Groweiss A., Kashman Y. (1981). Sipholenol and sipholenone, two new triterpenes from the marine sponge siphonochalina siphonella (levi). Tetrahedron Lett..

[51] Carmely S., Kashman Y. (1983). The sipholanes, a novel group of triterpenes from the marine sponge Siphonochalina siphonella. J. Org. Chem..

[52] Carmely S., Loya Y., Kashman Y. (1983). Siphenellinol, a new triterpene from the marine sponge siphonochalinasiphonella. Tetrahedron Lett..

[53] Kashman Y., Yosief T., Carmeli S. (2001). New Triterpenoids from the Red Sea Sponge Siphonochalina siphonella. J. Nat. Prod..

[54] Jain S., Abraham I., Carvalho P., Kuang Y.-H., Shaala L.A., Youssef D.T.A., Avery M.A., Chen Z.-S., El Sayed K.A. (2009). Sipholane Triterpenoids: Chemistry, Reversal of ABCB1/P-Glycoprotein-Mediated Multidrug Resistance, and Pharmacophore Modeling. J. Nat. Prod..

[55] Al-Lihaibi S.S., Abdel-Lateff A., Alarif W.M., Nogata Y., Ayyad S.-E.N., Okino T. (2015). Potent Antifouling Metabolites from Red Sea Organisms. Asian J. Chem..

[56] Li Y.-X., Himaya S., Kim S.-K. (2013). Triterpenoids of Marine Origin as Anti-Cancer Agents. Molecules.

[57] Jain S., Laphookhieo S., Shi Z., Fu L.-W., Akiyama S.-I., Chen Z.-S., Youssef D.T.A., van Soest R.W.M., El Sayed K.A. (2007). Reversal of P-Glycoprotein-Mediated Multidrug Resistance by Sipholane Triterpenoids. J. Nat. Prod..

[58] Shi Z., Jain S., Kim I.-W., Peng X.-X., Abraham I., Youssef D.T.A., Fu L.-W., El Sayed K., Ambudkar S.V., Chen Z.-S. (2007). Sipholenol A, a marine-derived sipholane triterpene, potently reverses P-glycoprotein (ABCB1)-mediated multidrug resistance in cancer cells. Cancer Sci..

[59] Abraham I., Jain S., Wu C.-P., Khanfar M.A., Kuang Y., Dai C.-L., Shi Z., Chen X., Fu L., Ambudkar S.V. (2010). Marine sponge-derived sipholane triterpenoids reverse P-glycoprotein (ABCB1)-mediated multidrug resistance in cancer cells. Biochem. Pharmacol..

[60] Abraham I., El Sayed K., Chen Z.-S., Guo H. (2012). Current Status on Marine Products with Reversal Effect on Cancer Multidrug Resistance. Mar. Drugs.

[61] Abdel-Lateff A., Al-Abd Ahmed M., Alahdal Abdulrahman M., Alarif Walied M., Ayyad Seif-Eldin N., Al-Lihaibi Sultan S., Hegazy Mohamed E., Al Mohammadi A., Abdelghany Tamer M., Abdel-Naim Ashraf B. (2016). Antiproliferative effects of triterpenoidal derivatives, obtained from the marine sponge *Siphonochalina* sp. on human hepatic and colorectal cancer cells. Z. Naturforsch. C J. Biosci..

[62] Carmely S., Kashman Y. (1986). Neviotine-A, a new triterpene from the red sea sponge Siphonochalina siphonella. J. Org. Chem..

[63] Angawi R., Saqer E., Abdel-Lateff A., Badria F., Ayyad S.-E. (2014). Cytotoxic neviotane triterpene-type from the red sea sponge Siphonochalina siphonella. Pharmacogn. Mag..

[64] Rudi A., Goldberg I., Stein Z., Benayahu Y., Schleyer M., Kashman Y. (1993). Sodwanones A–C, three new triterpenoids from a marine sponge. Tetrahedron Lett..

[65] Rudi A., Kashman Y., Benayahu Y., Schleyer M. (1994). Sodwanones A–F, New Triterpenoids from the Marine Sponge Axinella weltneri. J. Nat. Prod..

[66] Rudi A., Stein Z., Goldberg I., Yosief T., Kashman Y., Schleyer M. (1998). Yardenone and abudinol two new triterpenes from the marine sponge Ptilocaulis spiculifer. Tetrahedron Lett..

[67] Carletti I., Long C., Funel C., Amade P. (2003). Yardenone A and B: New Cytotoxic Triterpenes from the Indian Ocean Sponge *Axinella* cf. bidderi. J. Nat. Prod..

[68] Rudi A., Aknin M., Gaydou E.M., Kashman Y. (1997). Sodwanones K, L, and M; New Triterpenes from the Marine Sponge Axinella weltneri. J. Nat. Prod..

[69] Rudi A., Goldberg I., Stein Z., Kashman Y., Benayahu Y., Schleyer M., Gravalos M.D.G. (1995). Sodwanones G, H, and I, New Cytotoxic Triterpenes from a Marine Sponge. J. Nat. Prod..

[70] Rudi A., Yosief T., Schleyer M., Kashman Y. (1999). Several new isoprenoids from two marine sponges of the family Axinellidae. Tetrahedron.

[71] Funel-Le Bon C., Berrué F., Thomas O.P., Reyes F., Amade P. (2005). Sodwanone S, a Triterpene from the Marine Sponge Axinella weltneri. J. Nat. Prod..

[72] Dai J., Fishback J.A., Zhou Y.-D., Nagle D.G. (2006). Sodwanone and Yardenone Triterpenes from a South African Species of the Marine Sponge Axinella Inhibit Hypoxia-Inducible Factor-1 (HIF-1) Activation in Both Breast and Prostate Tumor Cells. J. Nat. Prod..

[73] Whibley C.E., Keyzers R.A., Soper A.G., Davies-Coleman M.T., Samaai T., Hendricks D.T. (2005). Antiesophageal Cancer Activity from Southern African Marine Organisms. Ann. N. Y. Acad. Sci..

[74] Isaacs S., Kashman Y. (1992). Shaagrockol B and C; two hexaprenylhydroquinone disulfates from the red sea sponge toxiclona toxius. Tetrahedron Lett..

[75] Loya S., Tal R., Hizi A., Issacs S., Kashman Y., Loya Y. (1993). Hexaprenoid Hydroquinones, Novel Inhibitors of the Reverse Transcriptase of Human Immunodeficiency Virus Type 1. J. Nat. Prod..

[76] Kornprobst J.-M., Sallenave C., Barnathan G. (1998). Sulfated Compounds from Marine Organisms. Comp. Biochem. Physiol. B Biochem. Mol. Biol..

[77] Prinsep M.R., Rahman A.-U. (2003). Sulfur-Containing Natural Products from Marine Invertebrates. Studies in Natural Products Chemistry.

[78] Bokesch H.R., Stull A.C., Pannell L.K., McKee T.C., Boyd M.R. (2002). A new pentacyclic sulfated hydroquinone from the marine sponge *Haliclona* sp.. Tetrahedron Lett..

[79] Menhour B., Aclinou P., Pete J.-P. (2013). Synthesis of bicyclic oxepanes: An enantioselective approach to the western part of Shaagrockol C. Med. J. Chem..

[80] Puliti R., Trivellone E., Crispino A., Cimino G. (1991). Raspacionin, a New Tetracyclic Triterpenoid from the Sponge Raspaciona aculeata: A Structure Containing Disordered Solvent. Acta Crystallogr. C.

[81] Cimino G., Crispino A., Epifanio R.d.A., Madaio A., Mattia C.A., Mazzarella L., Puliti R., Enrico T., Maria U. (1992). Raspacionin-A: A novel rearranged triterpenoid from the mediterranean sponge raspaciona aculeata. Tetrahedron.

[82] Cimino G., Crispino A., Madaio A., Trivellone E., Uriz M. (1993). Raspacionin B, a Further Triterpenoid from the Mediterranean Sponge Raspaciona aculeata. J. Nat. Prod..

[83] Cimino G., Epifanio R.D.A., Madaio A., Puliti R., Trivellone E. (1993). Absolute Stereochemistry of Raspacionin, the Main Triterpenoid from the Marine Sponge Raspaciona Aculeata. J. Nat. Prod..

[84] Cimino G., Madaio A., Trivellone E., Uriz M. (1994). Minor Triterpenoids from the Mediterranean Sponge, Raspaciona aculeata. J. Nat. Prod..

[85] Ciavatta M.L., Scognamiglio G., Trivellone E., Bisogno T., Cimino G. (2002). New additional triterpenoids from the Mediterranean sponge Raspaciona aculeata. Tetrahedron.

[86] Horak R.M., Steyn P.S., Van Rooyen P.H., Vleggaar R., Rabie C.J. (1981). Structures of the austalides A–E, five noval toxic metabolites from Aspergillus ustus. J. Chem. Soc. Chem. Commun..

[87] Horak R.M., Steyn P.S., Vleggaar R., Rabie C.J. (1985). Metabolites of *Aspergillus ustus*. Part 3. Structure elucidation of austalides G–L. J. Chem. Soc. Perkin Trans. 1.

[88] Zhou Y., Mándi A., Debbab A., Wray V., Schulz B., Müller W.E.G., Lin W., Proksch P., Kurtán T., Aly A.H. (2011). New Austalides from the Sponge-Associated Fungus *Aspergillus* sp.. Eur. J. Org. Chem..

[89] Zhou Y., Debbab A., Wray V., Lin W., Schulz B., Trepos R., Pile C., Hellio C., Proksch P., Aly A.H. (2014). Marine bacterial inhibitors from the sponge-derived fungus *Aspergillus* sp.. Tetrahedron Lett..

[90] Zhuravleva O.I., Sobolevskaya M.P., Leshchenko E.V., Kirichuk N.N., Denisenko V.A., Dmitrenok P.S., Dyshlovoy S.A., Zakharenko A.M., Kim N.Y., Afiyatullov S.S. (2014). Meroterpenoids from the Alga-Derived Fungi Penicillium thomii Maire and Penicillium lividum Westling. J. Nat. Prod..

[91] Shan W.-G., Wu Z.-Y., Pang W.-W., Ma L.-F., Ying Y.-M., Zhan Z.-J. (2015). α-Glucosidase Inhibitors from the Fungus Aspergillus terreus 3.05358. Chem. Biodivers..

[92] Peng J., Zhang X., Wang W., Zhu T., Gu Q., Li D. (2016). Austalides S–U, New Meroterpenoids from the Sponge-Derived Fungus Aspergillus aureolatus HDN14-107. Mar. Drugs.

[93] De Jesus A.E., Horak R.M., Steyn P.S., Vleggaar R. (1983). Biosynthesis of austalide D, a meroterpenoid mycotoxin from Aspergillus ustus. J. Chem. Soc. Chem. Commun..

[94] De Jesus A.E., Horak R.M., Steyn P.S., Vleggaar R. (1987). Metabolites of Aspergillus ustus. Part 4. Stable-isotope labelling studies on the biosynthesis of the austalides. J. Chem. Soc. Perkin Trans. 1.

[95] Dillen J.L.M., Horak R.M., Maharaj V.J., Marais S.F., Vleggaar R. (1989). Absolute configuration and biosynthesis of the austalides, meroterpenoid metabolites of Aspergillus ustus: Mode of cyclisation of the farnesyl moiety. J. Chem. Soc. Chem. Commun..

[96] Paquette L.A., Wang T.-Z., Sivik M.R. (1994). Enantioselective synthesis of natural (−)-austalide B, an unusual ortho ester metabolite produced by toxigenic cultures of Aspergillus ustus. J. Am. Chem. Soc..

[97] Paquette L.A., Wang T.-Z., Sivik M.R. (1994). Total Synthesis of (−)-Austalide B. A Generic Solution to Elaboration of the Pyran/p-Cresol/Butenolide Triad. J. Am. Chem. Soc..

[98] Rotem M., Carmely S., Kashman Y., Loya Y. (1983). Two new antibiotics from the red sea sponge Psammaplysilla purpurea. Tetrahedron.

[99] Roll D.M., Chang C.W.J., Scheuer P.J., Gray G.A., Shoolery J.N., Matsumoto G.K., Van Duyne G.D., Clardy J. (1985). Structure of the psammaplysins. J. Am. Chem. Soc..

[100] Mándi A., Mudianta I.W., Kurtán T., Garson M.J. (2015). Absolute Configuration and Conformational Study of Psammaplysins A and B from the Balinese Marine Sponge Aplysinella strongylata. J. Nat. Prod..

[101] Copp B.R., Ireland C.M., Barrows L.R. (1992). Psammaplysin C: A New Cytotoxic Dibromotyrosine-Derived Metabolite from the Marine Sponge Druinella (=Psammaplysilla) purpurea. J. Nat. Prod..

[102] Ichiba T., Scheuer P.J., Kelly-Borges M. (1993). Three bromotyrosine derivatives, one terminating in an unprecedented diketocyclopentenylidene enamine. J. Org. Chem..

[103] Tsukamoto S., Kato H., Hirota H., Fusetani N. (1996). Ceratinamides A and B: New antifouling dibromotyrosine derivatives from the marine sponge Pseudoceratina purpurea. Tetrahedron.

[104] Liu S., Fu X., Schmitz F.J., Kelly-Borges M. (1997). Psammaplysin F, a New Bromotyrosine Derivative from a Sponge, *Aplysinella* sp.. J. Nat. Prod..

[105] Nicholas G.M., Eckman L.L., Ray S., Hughes R.O., Pfefferkorn J.A., Barluenga S., Nicolaou K.C., Bewley C.A. (2002). Bromotyrosine-Derived Natural and Synthetic Products as Inhibitors of Mycothiol-*S*-Conjugate Amidase. Bioorg. Med. Chem. Lett..

[106] Nicholas G.M., Eckman L.L., Newton G.L., Fahey R.C., Ray S., Bewley C.A. (2003). Inhibition and kinetics of mycobacterium tuberculosis and mycobacterium smegmatis mycothiol-S-conjugate amidase by natural product inhibitors. Bioorg. Med. Chem..

[107] Yang X., Davis R.A., Buchanan M.S., Duffy S., Avery V.M., Camp D., Quinn R.J. (2010). Antimalarial Bromotyrosine Derivatives from the Australian Marine Sponge *Hyattella* sp.. J. Nat. Prod..

[108] Xu M., Andrews K.T., Birrell G.W., Tran T.L., Camp D., Davis R.A., Quinn R.J. (2011). Psammaplysin H, a new antimalarial bromotyrosine alkaloid from a marine sponge of the genus Pseudoceratina. Bioorg. Med. Chem. Lett..

[109] Wright A.D., Schupp P.J., Schrör J.-P., Engemann A., Rohde S., Kelman D., de Voogd N., Carroll A., Motti C.A. (2012). Twilight Zone Sponges from Guam Yield Theonellin Isocyanate and Psammaplysins I and J. J. Nat. Prod..

[110] Mudianta I.W., Skinner-Adams T., Andrews K.T., Davis R.A., Hadi T.A., Hayes P.Y., Garson M.J. (2012). Psammaplysin Derivatives from the Balinese Marine Sponge Aplysinella strongylata. J. Nat. Prod..

[111] Lee Y.-J., Han S., Lee H.-S., Kang J.S., Yun J., Sim C.J., Shin H.J., Lee J.S. (2013). Cytotoxic Psammaplysin Analogues from a Suberea sp. Marine Sponge and the Role of the Spirooxepinisoxazoline in Their Activity. J. Nat. Prod..

[112] Berlinck R.G.S., Burtoloso A.C.B., Kossuga M.H. (2008). The chemistry and biology of organic guanidine derivatives. Nat. Prod. Rep..

[113] Ohtani I., Kusumi T., Kakisawa H., Kashman Y., Hirsh S. (1992). Structure and chemical properties of ptilomycalin A. J. Am. Chem. Soc..

[114] Kashman Y., Hirsh S., McConnell O.J., Ohtani I., Kusumi T., Kakisawa H. (1989). Ptilomycalin A: A novel polycyclic guanidine alkaloid of marine origin. J. Am. Chem. Soc..

[115] Jares-Erijman E.A., Sakai R., Rinehart K.L. (1991). Crambescidins: New antiviral and cytotoxic compounds from the sponge Crambe crambe. J. Org. Chem..

[116] Jares-Erijman E.A., Ingrum A.L., Carney J.R., Rinehart K.L., Sakai R. (1993). Polycyclic guanidine-containing compounds from the Mediterranean sponge Crambe crambe: The structure of 13,14,15-isocrambescidin 800 and the absolute stereochemistry of the pentacyclic guanidine moieties of the crambescidins. J. Org. Chem..

[117] Palagiano E., De Marino S., Minale L., Riccio R., Zollo F., Iorizzi M., Carré J.B., Debitus C., Lucarain L., Provost J. (1995). Ptilomycalin A, crambescidin 800 and related new highly cytotoxic guanidine alkaloids from the starfishes Fromia monilis and Celerina heffernani. Tetrahedron.

[118] Venkateswarlu Y., Reddy M.V.R., Ramesh P., Rao J.V. (1999). Neofolitispates, pentacyclic guanidine alkaloids from the sponge Neofolitispa dianchora. Indian J. Chem..

[119] Braekman J.C., Daloze D., Tavares R., Hajdu E., Van Soest R.W.M. (2000). Novel Polycyclic Guanidine Alkaloids from Two Marine Sponges of the Genus Monanchora. J. Nat. Prod..

[120] Makarieva T.N., Tabakmaher K.M., Guzii A.G., Denisenko V.A., Dmitrenok P.S., Shubina L.K., Kuzmich A.S., Lee H.-S., Stonik V.A. (2011). Monanchocidins B–E: Polycyclic Guanidine Alkaloids with Potent Antileukemic Activities from the Sponge Monanchora pulchra. J. Nat. Prod..

[121] Shi Y., Moazami Y., Pierce J.G. (2017). Structure, synthesis and biological properties of the pentacyclic guanidinium alkaloids. Bioorg. Med. Chem..

[122] Lazaro J.E.H., Nitcheu J., Mahmoudi N., Ibana J.A., Mangalindan G.C., Black G.P., Howard-Jones A.G., Moore C.G., Thomas D.A., Mazier D. (2006). Antimalarial Activity of Crambescidin 800 and Synthetic Analogues against Liver and Blood Stage of *Plasmodium* sp.. J. Antibiot..

[123] Rubiolo J., Ternon E., López-Alonso H., Thomas O., Vega F., Vieytes M., Botana L. (2013). Crambescidin-816 Acts as a Fungicidal with More Potency than Crambescidin-800 and -830, Inducing Cell Cycle Arrest, Increased Cell Size and Apoptosis in Saccharomyces cerevisiae. Mar. Drugs.

[124] Aoki S., Kong D., Matsui K., Kobayashi M. (2004). Erythroid Differentiation in K562 Chronic Myelogenous Cells Induced by Crambescidin 800, a Pentacyclic Guanidine Alkaloid. Anticancer Res..

[125] Aron Z.D., Pietraszkiewicz H., Overman L.E., Valeriote F., Cuevas C. (2004). Synthesis and anticancer activity of side chain analogs of the crambescidin alkaloids. Bioorg. Med. Chem. Lett..

[126] Mayer A.M.S., Gustafson K.R. (2006). Marine pharmacology in 2003–2004: Anti-tumour and cytotoxic compounds. Eur. J. Cancer.

[127] Rubiolo J.A., López-Alonso H., Roel M., Vieytes M.R., Thomas O., Ternon E., Vega F.V., Botana L.M. (2014). Mechanism of cytotoxic action of crambescidin-816 on human liver-derived tumour cells. Br. J. Pharmacol..

[128] Mendez A.G., Juncal A.B., Silva S.B.L., Thomas O.P., Martín Vázquez V., Alfonso A., Vieytes M.R., Vale C., Botana L.M. (2017). The Marine Guanidine Alkaloid Crambescidin 816 Induces Calcium Influx and Cytotoxicity in Primary Cultures of Cortical Neurons through Glutamate Receptors. ACS Chem. Neurosci..

[129] Berlinck R.G.S., Braekman J.C., Daloze D., Bruno I., Riccio R., Ferri S., Spampinato S., Speroni E. (1993). Polycyclic Guanidine Alkaloids from the Marine Sponge Crambe crambe and Ca++ Channel Blocker Activity of Crambescidin 816. J. Nat. Prod..

[130] Ohizumi Y., Sasaki S., Kusumi T., Ohtani I.I. (1996). Ptilomycalin A, a novel Na(+), K(+)- or Ca2(+)-ATPase inhibitor, competitively interacts with ATP at its binding site. Eur. J. Pharmacol..

[131] Suna H., Aoki S., Setiawan A., Kobayashi M. (2007). Crambescidin 800, a pentacyclic guanidine alkaloid, protects a mouse hippocampal cell line against glutamate-induced oxidative stress. J. Nat. Med..

[132] Donahue M.G., Gribble G.W., Joule J.A. (2014). Chapter 1—Recent Developments in the Synthesis of Cyclic Guanidine Alkaloids. Progress in Heterocyclic Chemistry.

[133] Ma Y., De S., Chen C. (2015). Syntheses of cyclic guanidine-containing natural products. Tetrahedron.

[134] Murphy P.J., Williams H.L. (1994). Synthesis of a pentacyclic model of ptilomycalin A. J. Chem. Soc. Chem. Commun..

[135] Murphy P.J., Williams H.L., Hursthouse M.B., Malik K.M.A. (1994). Synthetic studies towards ptilomycalin a using A biomimetic approach. J. Chem. Soc. Chem. Commun..

[136] Black G.P., Murphy P.J., Walshe N.D.A., Hibbs D.E., Hursthouse M.B., Abdul Malik K.M. (1996). A short synthetic route to the tricyclic guanidinium core of the batzelladine alkaloids. Tetrahedron Lett..

[137] Murphy P.J., Harri Lloyd W., Hibbs D.E., Hursthouse M.B., Abdul Malik K.M. (1996). Biomimetic model studies towards ptilomycalin A. Tetrahedron.

[138] Murphy P.J., Williams H.L., Hibbs D.E., Hursthouse M.B., Malik K.M.A. (1996). Crystallographic evidence for the proposed host behaviour of ptilomycalin A. Chem. Commun..

[139] Black G.P., Murphy P.J., Walshe N.D.A. (1998). A short synthetic route to the tricyclic guanidinium core of the batzelladine alkaloids. Tetrahedron.

[140] Black G.P., Murphy P.J., Thornhill A.J., Walshe N.D.A., Zanetti C. (1999). Synthesis of the left hand unit of batzelladine F; Revision of the reported relative stereochemistry. Tetrahedron.

[141] Overman L.E., Rabinowitz M.H., Renhowe P.A. (1995). Enantioselective Total Synthesis of (−)-Ptilomycalin A. J. Am. Chem. Soc..

[142] Coffey D.S., McDonald A.I., Overman L.E., Rabinowitz M.H., Renhowe P.A. (2000). A Practical Entry to the Crambescidin Family of Guanidine Alkaloids. Enantioselective Total Syntheses of Ptilomycalin A, Crambescidin 657 and Its Methyl Ester (Neofolitispates 2), and Crambescidin 800. J. Am. Chem. Soc..

[143] Coffey D.S., Overman L.E., Stappenbeck F. (2000). Enantioselective Total Syntheses of 13,14,15-Isocrambescidin 800 and 13,14,15-Isocrambescidin 657. J. Am. Chem. Soc..

[144] Overman L.E., Rhee Y.H. (2005). Total Synthesis of (−)-Crambidine and Definition of the Relative Configuration of Its Unique Tetracyclic Guanidinium Core. J. Am. Chem. Soc..

[145] Perl N.R., Ide N.D., Prajapati S., Perfect H.H., Durón S.G., Gin D.Y. (2010). Annulation of Thioimidates and Vinyl Carbodiimides to Prepare 2-Aminopyrimidines, Competent Nucleophiles for Intramolecular Alkyne Hydroamination. Synthesis of (−)-Crambidine. J. Am. Chem. Soc..

[146] Nagasawa K., Georgieva A., Koshino H., Nakata T., Kita T., Hashimoto Y. (2002). Total Synthesis of Crambescidin 359. Org. Lett..

[147] Moore C.G., Murphy P.J., Williams H.L., McGown A.T., Smith N.K. (2003). A synthesis of crambescidin 359. Tetrahedron Lett..

[148] Aron Z.D., Overman L.E. (2005). Total Synthesis and Properties of the Crambescidin Core Zwitterionic Acid and Crambescidin 359. J. Am. Chem. Soc..

[149] Gomes N., Lefranc F., Kijjoa A., Kiss R. (2015). Can Some Marine-Derived Fungal Metabolites Become Actual Anticancer Agents?. Mar. Drugs.

[150] Cutler H.G., Springer J.P., Arrendale R.F., Arison B.H., Cole P.D., Roberts R.G. (1988). Cinereain: A Novel Metabolite with Plant Growth Regulating Properties from Botrytis cinerea. Agric. Biol. Chem..

[151] Rahbæk L., Breinholt J., Frisvad J.C., Christophersen C. (1999). Circumdatin A, B, and C: Three New Benzodiazepine Alkaloids Isolated from a Culture of the Fungus Aspergillus ochraceus. J. Org. Chem..

[152] Ookura R., Kito K., Ooi T., Namikoshi M., Kusumi T. (2008). Structure Revision of Circumdatins A and B, Benzodiazepine Alkaloids Produced by Marine Fungus Aspergillus ostianus, by X-ray Crystallography. J. Org. Chem..

[153] González-Jartín J.M., Alfonso A., Sainz M.J., Vieytes M.R., Botana L.M. (2017). UPLC-MS-IT-TOF Identification of Circumdatins Produced by Aspergillus ochraceus. J. Agric. Food. Chem..

[154] Belofsky G.N., Anguera M., Jensen P.R., Fenical W., Köck M. (2000). Oxepinamides A–C and Fumiquinazolines H–I: Bioactive Metabolites from a Marine Isolate of a Fungus of the Genus Acremonium. Chem. Eur. J..

[155] Sprogøe K., Manniche S., Larsen T.O., Christophersen C. (2005). Janoxepin and brevicompanine B: Antiplasmodial metabolites from the fungus Aspergillus janus. Tetrahedron.

[156] Doveston R.G., Steendam R., Jones S., Taylor R.J.K. (2012). Total Synthesis of an Oxepine Natural Product, (±)-Janoxepin. Org. Lett..

[157] Lee S.U., Asami Y., Lee D., Jang J.-H., Ahn J.S., Oh H. (2011). Protuboxepins A and B and Protubonines A and B from the Marine-Derived Fungus *Aspergillus* sp. SF-5044. J. Nat. Prod..

[158] Asami Y., Jang J.-H., Soung N.-K., He L., Moon D.O., Kim J.W., Oh H., Muroi M., Osada H., Kim B.Y. (2012). Protuboxepin A, a marine fungal metabolite, inducing metaphase arrest and chromosomal misalignment in tumor cells. Bioorg. Med. Chem..

[159] Zhang P., Mándi A., Li X.-M., Du F.-Y., Wang J.-N., Li X., Kurtán T., Wang B.-G. (2014). Varioxepine A, a 3H-Oxepine-Containing Alkaloid with a New Oxa-Cage from the Marine Algal-Derived Endophytic Fungus Paecilomyces variotii. Org. Lett..

[160] Zhang P., Li X.-M., Wang J.-N., Wang B.-G. (2015). Oxepine-Containing Diketopiperazine Alkaloids from the Algal-Derived Endophytic Fungus Paecilomyces variotii EN-291. Helv. Chim. Acta.

[161] Wang J., He W., Huang X., Tian X., Liao S., Yang B., Wang F., Zhou X., Liu Y. (2016). Antifungal New Oxepine-Containing Alkaloids and Xanthones from the Deep-Sea-Derived Fungus Aspergillus versicolor SCSIO 05879. J. Agric. Food. Chem..

[162] Pan C., Shi Y., Chen X., Chen C.-T.A., Tao X., Wu B. (2017). New compounds from a hydrothermal vent crab-associated fungus Aspergillus versicolor XZ-4. Org. Biomol. Chem..

[163] Gardiner D.M., Waring P., Howlett B.J. (2005). The epipolythiodioxopiperazine (ETP) class of fungal toxins: distribution, mode of action, functions and biosynthesis. Microbiology.

[164] Isaka M., Kittakoop P., Kirtikara K., Hywel-Jones N.L., Thebtaranonth Y. (2005). Bioactive Substances from Insect Pathogenic Fungi. Acc. Chem. Res..

[165] Borthwick A.D. (2012). 2,5-Diketopiperazines: Synthesis, Reactions, Medicinal Chemistry, and Bioactive Natural Products. Chem. Rev..

[166] Munday R. (2012). Harmful and Beneficial Effects of Organic Monosulfides, Disulfides, and Polysulfides in Animals and Humans. Chem. Res. Toxicol..

[167] Bladt T., Frisvad J., Knudsen P., Larsen T. (2013). Anticancer and Antifungal Compounds from Aspergillus, Penicillium and Other Filamentous Fungi. Molecules.

[168] Huang R.-M., Yi X.-X., Zhou Y., Su X., Peng Y., Gao C.-H. (2014). An Update on 2,5-Diketopiperazines from Marine Organisms. Mar. Drugs.

[169] Kuppusamy P., Yusoff M.M., Maniam G.P., Ichwan S.J.A., Soundharrajan I., Govindan N. (2014). Nutraceuticals as potential therapeutic agents for colon cancer: A review. Acta Pharm. Sin. B.

[170] Welch T.R., Williams R.M. (2014). Epidithiodioxopiperazines. occurrence, synthesis and biogenesis. Nat. Prod. Rep..

[171] Nadumane V.K., Venkatachalam P., Gajaraj B., Vijai G. (2016). Chapter 19—Aspergillus Applications in Cancer Research A2. New and Future Developments in Microbial Biotechnology and Bioengineering.

[172] Nagarajan R., Huckstep L.L., Lively D.H., DeLong D.C., Marsh M.M., Neuss N. (1968). Aranotin and related metabolites from Arachniotus aureus. I. Determination of structure. J. Am. Chem. Soc..

[173] Neuss N., Nagarajan R., Molloy B.B., Huckstep L.L. (1968). Aranotin and related metabolites. II. Isolation, characterization, and structures of two new metabolites. Tetrahedron Lett..

[174] Nagarajan R., Woody R.W. (1973). Circular dichroism of gliotoxin and related epidithiapiperazinediones. J. Am. Chem. Soc..

[175] Murdock K.C. (1974). Antiviral agents. Chemical modifications of a disulfide antibiotic, acetylaranotin. J. Med. Chem..

[176] Kamata S., Sakai H., Hirota A. (1983). Isolation of Acetylaranotin, Bisdethiodi(methylthio)-acetylaranotin and Terrein as Plant Growth Inhibitors from a Strain of Aspergillus terreus. Agric. Biol. Chem..

[177] Guo C.-J., Yeh H.-H., Chiang Y.-M., Sanchez J.F., Chang S.-L., Bruno K.S., Wang C.C.C. (2013). Biosynthetic Pathway for the Epipolythiodioxopiperazine Acetylaranotin in Aspergillus terreus Revealed by Genome-Based Deletion Analysis. J. Am. Chem. Soc..

[178] Choi E.J., Park J.S., Kim Y.J., Jung J.H., Lee J.K., Kwon H.C., Yang H.O. (2011). Apoptosis-inducing effect of diketopiperazine disulfides produced by *Aspergillus* sp. KMD 901 isolated from marine sediment on HCT116 colon cancer cell lines. J. Appl. Microbiol..

[179] Seya H., Nakajima S., Kawai K.-I., Udagawa S.-I. (1985). Structure and absolute configuration of emestrin, a new macrocyclic epidithiodioxopiperazine from Emericell striata. J. Chem. Soc. Chem. Commun..

[180] Seya H., Nozawa K., Nakajima S., Kawai K.-I., Udagawa S.-I. (1986). Studies on fungal products. Part 8. Isolation and structure of emestrin, a novel antifungal macrocyclic epidithiodioxopiperazine from Emericella striata. X-ray molecular structure of emestrin. J. Chem. Soc. Perkin Trans. 1.

[181] Seya H., Nozawa K., Udagawa S.-I., Nakajima S., Kawai K.-I. (1986). Studies on Fungal Products. IX. Dethiosecoemestrin, a New Metabolite Related to Emestrin, from Emericella striata. Chem. Pharm. Bull..

[182] Ooike M., Nozawa K., Kawai K.-I. (1997). An epitetrathiodioxopiperazine related to emestrin from Emericella foveolata. Phytochemistry.

[183] Kawahara N., Nakajima S., Yamazaki M., Kawai K. (1989). Structure of a Novel Epidithiodioxopiperazine, Emethallicin A, a Potent Inhibitor of Histamine Release, from Emericella heterothallica. Chem. Pharm. Bull..

[184] Kawahara N., Nozawa K., Yamazaki M., Nakajima S., Kawai K.-I. (1990). Structures of Novel Epipolythiodioxopiperazines, Emethallicins B, C, and D, Potent Inhibitors of Histamine Release, from Emericella heterothallica. Chem. Pharm. Bull..

[185] Goodman R.M., Kishi Y. (1998). Experimental Support for the Primary Stereoelectronic Effect Governing Baeyer–Villiger Oxidation and Criegee Rearrangement. J. Am. Chem. Soc..

[186] Peng J., Clive D.L.J. (2007). Synthesis of Dihydrooxepin Models Related to the Antitumor Antibiotic MPC1001. Org. Lett..

[187] Gross U., Nieger M., Bräse S. (2010). A Unified Strategy Targeting the Thiodiketopiperazine Mycotoxins Exserohilone, Gliotoxin, the Epicoccins, the Epicorazines, Rostratin A and Aranotin. Chem. Eur. J..

[188] Nicolaou K.C., Lu M., Totokotsopoulos S., Heretsch P., Giguère D., Sun Y.-P., Sarlah D., Nguyen T.H., Wolf I.C., Smee D.F. (2012). Synthesis and Biological Evaluation of Epidithio-, Epitetrathio-, and bis-(Methylthio)diketopiperazines: Synthetic Methodology, Enantioselective Total Synthesis of Epicoccin G, 8,8′-epi-ent-Rostratin B, Gliotoxin, Gliotoxin G, Emethallicin E, and Haematocin and Discovery of New Antiviral and Antimalarial Agents. J. Am. Chem. Soc..

[189] Schuber P.T., Williams R.M. (2012). Synthetic studies toward MPC1001: Preparation of a β-hydroxyl-tyrosine derivative. Tetrahedron Lett..

[190] Zipfel H.F., Carreira E.M. (2014). An Efficient Synthesis Strategy to the Core Structure of 6-5-6-5-6-Membered Epipolythiodiketopiperazines. Org. Lett..

[191] Belov D.S., Ratmanova N.K., Andreev I.A., Kurkin A.V. (2015). Synthesis of Bicyclic Proline Derivatives by the Aza-Cope-Mannich Reaction: Formal Synthesis of (±)-Acetylaranotin. Chem. Eur. J..

[192] Zheng L., Liang Y.-M. (2017). Copper-Catalyzed [2 + 2 + 3] Annulation of 1,6-Enynes with α-Bromo-1,3-Dicarbonyl Compounds: Synthesis of Dihydrooxepines. J. Org. Chem..

[193] Codelli J.A., Puchlopek A.L.A., Reisman S.E. (2012). Enantioselective Total Synthesis of (−)-Acetylaranotin, a Dihydrooxepine Epidithiodiketopiperazine. J. Am. Chem. Soc..

[194] Onodera H., Hasegawa A., Tsumagari N., Nakai R., Ogawa T., Kanda Y. (2004). MPC1001 and Its Analogues: New Antitumor Agents from the Fungus Cladorrhinum Species. Org. Lett..

[195] Tsumaragi N., Nakai R., Onodera H., Hasegawa A., Rahayu E.S., Ando K., Yamashita Y. (2004). MPC1001, a New Antitumor Antibiotic Produced by *Cladorrhinum* sp.. J. Antibiot..

[196] Wang H., Regan C.J., Codelli J.A., Romanato P., Puchlopek-Dermenci A.L.A., Reisman S.E. (2017). Enantioselective Synthesis of (−)-Acetylapoaranotin. Org. Lett..

